# LFA-1/ICAM-1 Interactions Between CD8^+^ and CD4^+^ T Cells Promote CD4^+^ Th1-Dominant Differentiation and CD8^+^ T Cell Cytotoxicity for Strong Antitumor Immunity After Cryo-Thermal Therapy

**DOI:** 10.3390/cells14080620

**Published:** 2025-04-21

**Authors:** Yichen Yao, Zelu Zhang, Shicheng Wang, Junjun Wang, Yuankai Hao, Ke Wang, Ping Liu

**Affiliations:** School of Biomedical Engineering and Med-X Research Institute, Shanghai Jiao Tong University, Shanghai 200030, China; yichenyao@sjtu.edu.cn (Y.Y.); zeluzhang1126@sjtu.edu.cn (Z.Z.); shichengwang@sjtu.edu.cn (S.W.); sjtuwangjunjun@sjtu.edu.cn (J.W.); haoyuankai@sjtu.edu.cn (Y.H.); k.0522@sjtu.edu.cn (K.W.)

**Keywords:** CD4^+^ T cells, CD8^+^ T cells, LFA-1, ICAM-1, Notch1, IL-2, cryo-thermal therapy

## Abstract

CD4^+^ T cells have been well-regarded as “helper” cells in activating the cytotoxicity of CD8^+^ T cells for effective tumor eradication, while few studies have focused on whether CD8^+^ T cells regulate CD4^+^ T cells. Our previous studies provided evidence for an interaction between CD4^+^ and CD8^+^ T cells after cryo-thermal therapy, but the mechanism remains unclear, especially pertaining to how CD8^+^ T cells promote the Th1 differentiation of CD4^+^ T cells. This study revealed that activated CD4^+^ and CD8^+^ T cells are critical for CTT-induced antitumor immunity, and the interaction between activated T cells is enhanced. The reciprocal regulation of activated CD8^+^ and CD4^+^ T cells was through LFA-1/ICAM-1 interactions, in which CD8^+^ T cells facilitate Notch1-dependent CD4^+^ Th1-dominant differentiation and promote IL-2 secretion of CD4^+^ T cells. Meanwhile, IL-2 derived from CD4^+^ T cells enhances the cytotoxicity of CD8^+^ T cells and establishes a positive feedback loop via increasing the expression of LFA-1 and ICAM-1 on T cells. Clinical analyses further validated that LFA-1/ICAM interactions between CD4^+^ and CD8^+^ T cells are correlated with clinical outcomes. Our study extends the functions of the LFA-1/ICAM-1 adhesion pathway, indicating its novel role in the interaction of CD4^+^ and CD8^+^ T cells.

## 1. Introduction

T lymphocytes have been extensively recognized for their critical role in antitumor immunity [1]. CD8^+^ cytotoxic T lymphocytes (CTLs) are considered the main executors of adaptive immunity, as they eliminate tumor cells by secreting death-inducing granules, such as granzymes/perforin, and modulating the Fas/FasL death receptor pathway [2,3]. CD4^+^ T cells are known as helper cells, as they stimulate effector CD8^+^ T cell responses directly, by secreting IFN-γ and IL-2, and indirectly, through CD40/CD40L-dependent dendritic cell (DC) activation [4,5,6]. Recent evidence has shown that the interactions between CD4^+^ T cells, CD8^+^ T cells, and DCs promote the formation of triads, creating a stable structure for CD4^+^ T cells to aid in preventing the terminal differentiation of CD8^+^ T cells by transcriptional and epigenetic reprogramming [7,8]. Numerous clinical studies have indicated that extensive infiltration of both CD4^+^ and CD8^+^ T cells is correlated with favorable prognosis in multiple cancer types [9,10,11]. Consequently, the coordinated activation of both CD4^+^ and CD8^+^ T cells constitutes a central determinant of effective antitumor immunity.

Cryo-thermal therapy (CTT) is a novel tumor ablation approach developed by our research team that has been approved by the China National Medicine Products Administration (no. 20233010773) and can achieve complete eradication of solid tumor tissues by combining precooling and radiofrequency ablation [12]. CTT rapidly disrupts tumor cells through a large thermomechanical stress, which induces immunogenic cell death in tumor cells and triggers the release of large amounts of tumor-associated antigens and damage-associated molecular patterns (DAMPs), to promote the maturation of M1 macrophages and DCs [13,14]. Furthermore, mature antigen-presenting cells (APCs) promote the cytotoxicity of CD8^+^ T cells and orchestrate the Th1-dominant differentiation of CD4^+^ T cells in order to sustain long-term antitumor immunity [15]. Our previous studies demonstrated that CTT-induced CD4^+^ T cells augment the expression of cytotoxic molecules in CD8^+^ T cells via IFN-γ and direct cell–cell contact [16]. Intriguingly, the Th1-dominant differentiation of CD4^+^ T cells is severely compromised by the absence of CTT-induced CD8^+^ T cells [16], which provides evidence for an interaction between CD4^+^ and CD8^+^ T cells. Moreover, little is known about the underlying mechanisms by which CD8^+^ T cells affect the differentiation of CD4^+^ T cells.

In this study, we established a B16F10 melanoma model to elucidate the interactions between CD4^+^ and CD8^+^ T cells during the priming phase of CTT-induced Th1-dominant differentiation. Anti-CD4 or anti-CD8α monoclonal antibodies were used to neutralize CD4^+^ or CD8^+^ T cells in vivo, and RNA-seq, in vivo and in vitro experiments, and clinical data analysis were used to investigate the interactions between CD4^+^ and CD8^+^ T cells. We revealed that CTT induced the activation of CD4^+^ and CD8^+^ T cells, which was necessary for the long-term survival of mice. Moreover, the aggregations of and interactions between activated CD4^+^ and CD8^+^ T cells were enhanced, with CD8^+^ T cells promoting CD4^+^ Th1-dominant differentiation and CD4^+^ T cells enhancing the cytotoxicity of CD8^+^ T cells. Mechanistically, CD8^+^ T cells interacted with CD4^+^ T cells via LFA-1/ICAM-1, which activated Notch1 signaling in CD4^+^ T cells to induce Th1 differentiation, and increased IL-2 secretion from CD4^+^ T cells. IL-2 derived from CD4^+^ T cells further augmented the expression of cytotoxic molecules in CD8^+^ T cells, which concomitantly increased the expression of LFA-1/ICAM-1 on T cells, thereby amplifying the interactions between CD4^+^ and CD8^+^ T cells. Our findings reveal a novel mechanism through which CD4^+^ and CD8^+^ T cell interactions contribute to sustained antitumor immunity after CTT.

## 2. Materials and Methods

### 2.1. Cell Culture and Animal Model

The B16F10 murine melanoma cell line was provided by Prof. Weihai Ying from the Shanghai Jiao Tong University‘s Med-X Research Institute, and the 4T1 murine breast cancer cells were obtained from Shanghai First People’s Hospital. These cell lines were cultured in DMEM (Meilunbio, Dalian, China) supplemented with 10% FBS (Gemini Bio-Products, West Sacramento, CA, USA) and 1% penicillin–streptomycin (Hyclone, Logan, UT, USA), and incubated at 37 °C with 5% CO_2_.

Female C57BL/6 and BALB/c mice, aged 6–8 weeks, were purchased from Shanghai Slaccas Experimental Animal Co., Ltd. (Shanghai, China) and kept in regular circumstances with a 12 h light/dark cycle, and sterile food and water. To establish tumor models, C57BL/6 mice received a subcutaneous injection of 5 × 10^5^ B16F10 cells in the right flank, while BALB/c mice were inoculated with 4 × 10^5^ 4T1 cells in the right flank. Mice were divided into cages at random and administered various treatments. All of the animal experiments were carried out in compliance with the rules set forth by Shanghai Jiao Tong University’s Animal Welfare Committee and the procedures authorized by the Shanghai Jiao Tong University Scientific Ethics Committee.

### 2.2. Cryo-Thermal Therapy Procedures

CTT is a local treatment approach which was invented by our laboratory. When tumors reached approximately 1 cm in diameter (day 12 or 16 post-inoculation), mice were randomly grouped to receive CTT. The process of CTT involves freezing mouse subcutaneous tumors in liquid nitrogen at −20 °C for five minutes, followed by radiofrequency heating at 50 °C for ten minutes.

### 2.3. CD4^+^ and CD8^+^ T Cell Depletion

Mice in the CTT group received intraperitoneal injections of a 300 μg Ultra-LEAF™ purified anti-mouse CD4 (Biolegend, San Diego, CA, USA, Clone GK 1.5) or CD8a (Biolegend, San Diego, CA, USA, Clone 53–6.7) antibody at day 5 post-CTT to deplete CD4^+^ or CD8^+^ T cells.

### 2.4. Flow Cytometry Analysis

To obtain single-cell suspensions, spleens and blood were collected from different groups at day 7 or 14 after CTT. Subsequently, the absolute cell counts were normalized by adding counting beads (Biolegend, San Diego, CA, USA, CAT# 424902). Erythrocyte lysis reagent, which contained 1.0 M of KHCO_3_, 0.15 M of NH_4_Cl, and 0.1 mM of Na_2_EDTA, was utilized to lyse red blood cells. The cell suspensions were filtered through 70 μm strainers. Zombie Aqua (BioLegend, San Diego, CA, USA) and fluorochrome-conjugated antibodies were employed to stain the cells (Table S1). The cells were treated with antibodies for 15 min at room temperature in order to mark surface proteins. Following 4–6 h of stimulation with a Cell Activation Cocktail (Biolegend, San Diego, CA, USA), surface markers staining, fixation with Fixation Buffer (Biolegend, San Diego, CA, USA), and permeabilization with Intracellular Staining Perm Wash Buffer (BioLegend, San Diego, CA, USA), the cells were stained for 20 min with intracellular cytokine antibodies in order to detect intracellular cytokine. The True-Nuclear™ Transcription Factor Buffer Set (Thermo Fisher Scientific, Waltham, MA, USA) and transcription factor antibodies were utilized for transcription factor staining. Cell fluorescence was measured using LSR Fortessa flow cytometry (BD Biosciences, Franklin Lakes, NJ, USA) and FlowJo (v10) was used for analysis, with the gating strategy detailed in Figure S1.

### 2.5. CD4^+^ and CD8^+^ T Cell Isolation

Spleens from different groups were gathered at different times, and single-cell suspensions were made for cell isolation. EasySep Mouse CD4 Positive Selection Kit II (StemCell Technologies, Vancouver, BC, Canada, CAT# 18952) and EasySep Mouse CD8a Positive Selection Kit II (StemCell Technologies, Vancouver, BC, Canada, CAT# 18753) were used to separate CD4^+^ and CD8^+^ T cells from splenocytes. Every cell isolation procedure was carried out in accordance with the manufacturer’s guidelines. Cell purity exceeded 90%.

### 2.6. In Vitro Tumor Killing Assay

Calcein-AM-labeled B16F10 tumor cells were cocultured with CD8^+^ T cells (separated from the control, CTT, and CTT with CD4^+^ T cell depletion groups) at ratios of 8:1, 16:1, and 32:1, or 2:1, 4:1, and 16:1, in 96-well V-bottom plates. After 6 h, supernatants were transferred to 96-well flat-bottomed black plates, and the fluorescence intensity was detected by an enzyme-labeled instrument (SpectraMax-i3, Molecular Devices, San Jose, CA, USA).

### 2.7. CD4^+^ and CD8^+^ T Cells Coculture System

CD4^+^ and CD8^+^ T lymphocytes extracted from CTT-treated mice on the fifth day post-CTT were cocultured at a 2:1 effector-to-target ratio for 24 h. The T cells were stimulated using the anti-CD3a antibody (1 μg/mL, Biolegend, San Diego, CA, USA, Clone 145-2C11). To explore the interaction mechanism between CD4^+^ and CD8^+^ T cells, the cells were separately cultured in 0.4 μm Transwell chambers, or the following were added: CD11a (LFA-1alpha) monoclonal antibody (5 μg/mL, Thermo Fisher Scientific, Waltham, MA, USA, Clone M17/4), CD54 (ICAM-1) monoclonal antibody (10 μg/mL, Thermo Fisher Scientific, Waltham, MA, USA, Clone YN1/1.7.4), recombinant mouse IL-2 (100 IU/mL, novoprotein, Suzhou, China, P04351), InVivoMAb anti-mouse IL-2 (15 μg/mL, Bioxcell, Lebanon, NH, USA, Clone JES6-5H4), STAT5 inhibitor BD750 (10 μM, MedChemExpress, Monmouth Junction, NJ, USA), or Notch1 signaling inhibitor DAPT (10 μM, MedChemExpress, Monmouth Junction, NJ, USA).

### 2.8. In Vivo LFA-1 Blockade

To block LFA-1/ICAM-1 signaling in vivo, mice received intraperitoneal injections of 150 μg InVivoMAb anti-mouse LFA-1α (CD11a) antibody (Bioxcell, Lebanon, NH, USA, Clone M17/4) every 12 h for three consecutive doses, starting at day 5 after CTT.

### 2.9. RNA-Seq and Analysis

Total RNA extraction from the splenic CD4^+^ and CD8^+^ T cells was conducted with TRIzol (Invitrogen, Waltham, MA, USA), and the quality and integrity of Total RNA were assessed using a NanoDrop 2000 spectrophotometer (Thermo Scientific, Waltham, MA, USA) and Agilent 2100 Bioanalyzer (Agilent Technologies, Santa Clara, CA, USA). The VAHTS Universal V6 RNA-seq Library Preparation Kit was then used to create RNA-seq libraries, which were then sequenced by OE Biotech Co., Ltd. (Shanghai, China). To pinpoint differentially expressed genes, we used DESeq2, and then GSEA based on GO, KEGG, Hallmark, and Wikipathways was performed using GSEA software (v4.3.2) and OECloud tools (https://cloud.oebiotech.com/task/ (accessed on 16 March 2025)).

### 2.10. Immunofluorescence Staining

Spleens were fixed in 10% neutral formaldehyde for 24 h, dehydrated, and then embedded in paraffin. The tissue slices were then obtained using a pathology sectioning machine (Leica, Shanghai, China). Dewaxing and hydration were performed on the tissue sections. Subsequently, antigen repair fluid (Tris-EDTA, Sigma-Aldrich, Saint Louis, MO, USA) was used for heat-induced antigen retrieval. Then, slides were blocked for 1 h at room temperature using 2% BSA (Sigma-Aldrich, Saint Louis, MO, USA), followed by an overnight incubation at 4 °C with anti-CD4 or anti-CD8a antibodies. The next day, slides were treated for 1 h at 37 °C using a goat anti-rabbit fluorescent antibody (Abcam, Cambridge, UK). Finally, sections were sealed with DAPI (Abcam, Cambridge, UK), and images were acquired with Nikon Eclipse Ci-L (Nikon, Tokyo, Japan).

### 2.11. IL-2 ELISA

The supernatant of the CD4^+^ and CD8^+^ T cell cocultured group, the Transwell group, and the LFA-1 blocking group were collected, and IL-2 levels were analyzed using the ELISA kit (Epizyme Biotech, Shanghai, China, CAT# HJ178). Refer to the instruction manual of the ELISA kit for detailed procedures.

### 2.12. Western Blot

CD4^+^ T cells were ruptured on ice for 1 h in 1×RIPA lysis buffer (Thermo Fisher Scientific, Waltham, MA, USA) which contained phosphatase inhibitor (Beyotime, Shanghai, China) and proteinase inhibitor (Beyotime, Shanghai, China). After being separated by 7.5% SDS-PAGE, proteins (10 μg per lane) were shuffled over to a PVDF membrane. This work made use of anti-Cleaved Notch1 (NICD) (Cell Signaling Technology, Danvers, MA, USA, CAT#4147), anti-β-actin (Cell Signaling Technology, Danvers, MA, USA, CAT#4967), and HRP-conjugated secondary antibody (EpiZyme Biotech, Shanghai, China, CAT#LF102). These images are representative of three independent experiments.

### 2.13. scRNA-Seq Analysis

The melanoma scRNA-seq dataset files of Sade-Feldman et al. [17] were downloaded from the 3CA database, including 32 melanoma patients with 48 tumor biopsies. The Seurat (v4.2.0) was used to analyze and process the gene expression matrix. The data were normalized and scaled with the “NormalizeData” and “ScaleData” commands, followed by PCA and cluster analysis. Visualization was achieved using the UMAP method. T cell cluster transcriptional profiles were characterized by identifying differentially expressed genes (DEGs) with the “FindMarkers” function. T cells subsets were annotated based on T cell type markers. The intercellular communication networks between CD4^+^ and CD8^+^ T cells were inferred through ligand-receptor analysis using the CellChat package (v1.6.1).

### 2.14. Statistical Analysis

The mean ± standard deviation (SD) was used to present all results. Comparisons were made using the Student’s *t*-test (two groups) or one-way ANOVA (multiple groups), and survival curves were analyzed using Kaplan–Meier analysis. The threshold for statistical significance was set at *p* < 0.05. For all analyses, GraphPad Prism 9.0 was used.

## 3. Results

### 3.1. CD4^+^ and CD8^+^ T Cells Were Activated at an Early Stage After CTT

**Figure 1:**
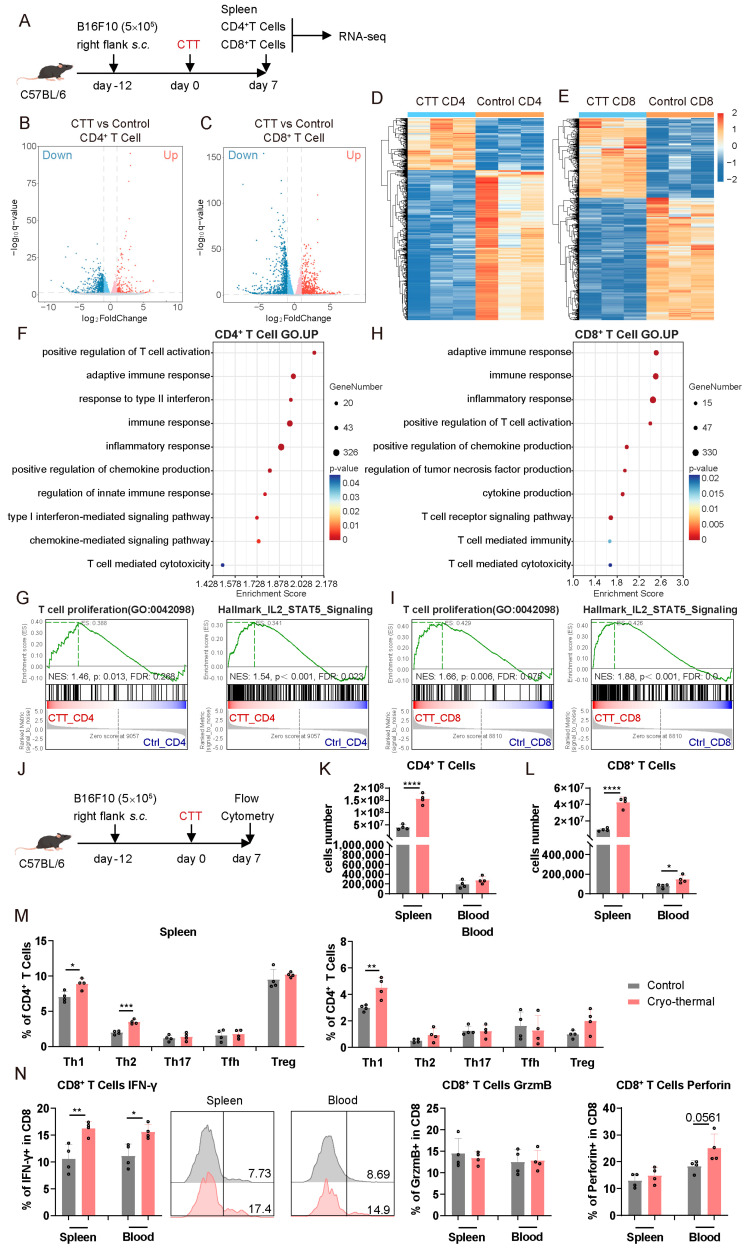
CD4^+^ and CD8^+^ T cells were activated and proliferated at an early stage after CTT. (**A**) Scheme of the experiment design. The splenic CD4^+^ and CD8^+^ T cells were isolated on day 7 after CTT by MACS for RNA-seq. (**B**,**C**) Volcano plot showing DEGs in (**B**) CD4^+^ and (**C**) CD8^+^ T cells from the control or CTT group. (**D**,**E**) Heatmap of differentially expressed genes in (**D**) CD4^+^ and (**E**) CD8^+^ T cells from the control or CTT group. (**F**) Scatter plot of the GO-enriched gene sets according to GSEA in CD4^+^ T cells. (**G**) Gene enrichment of T cell proliferation and IL2–STAT5 signaling pathways according to GSEA in CD4^+^ T cells. (**H**) Scatter plot of the GO-enriched gene sets according to GSEA in CD8^+^ T cells. (**I**) Gene enrichment of T cell proliferation and IL2–STAT5 signaling pathways according to GSEA in CD8^+^ T cells. (**J**) Scheme of the experiment design. The phenotype of CD4^+^ and CD8^+^ T cells was detected by flow cytometry on day 7 after CTT. (**K**,**L**) Absolute number of (**K**) CD4^+^ and (**L**) CD8^+^ T cells in the spleen and blood. (**M**) Subsets of CD4^+^ T cells in the spleen and blood. (**N**) IFN-γ, granzyme B, and perforin expression in CD8^+^ T cells in the spleen and blood. All of the data are presented as the means ± SD. n = 4 for flow cytometry and n = 3 for RNA-seq. * *p* < 0.05, ** *p* < 0.01, *** *p* < 0.001, **** *p* < 0.0001. Data for graphs were calculated by Student’s *t*-test.

To investigate the interactions between CD4^+^ and CD8^+^ T cells, we first assessed the changes of CD4^+^ and CD8^+^ T cells in a B16F10 tumor model early after CTT (day 7), during the priming phase of CTT-induced Th1-dominant differentiation. The gene expression profiles of splenic T cells from the tumor-bearing and CTT-treated mice were investigated using RNA-seq on day 7 after CTT (Figure 1A). Notably, both CD4^+^ and CD8^+^ T cells in the CTT group exhibited tremendous transcriptional alterations compared to those in the tumor-bearing group (Figure 1B–E). The gene set enrichment analysis (GSEA) based on GO terms revealed that pathways related to T cell-mediated inflammation, the immune response, cytotoxicity, and cytokine and chemokine production were substantially activated in both CD4^+^ and CD8^+^ T cells after CTT, indicating T cell activation (Figure 1F,H). Moreover, the T cell proliferation pathway and IL-2–STAT5 signaling pathway, which are associated with T cell survival and expansion, were enriched in CTT-induced CD4^+^ and CD8^+^ T cells (Figure 1G,I). Additionally, the Th1 and Th2 cell differentiation pathways identified using the KEGG database were also significantly enriched in CD4^+^ T cells after CTT (Figure S2A). However, GSEA based on KEGG terms revealed that the cell cycle and DNA replication pathways in both CD4^+^ and CD8^+^ T cells after CTT were downregulated (Figure S2B–E). Cell cycle arrest in T cells usually occurs with an overly robust immune response to balance antigen clearance with a hyperimmune response, and these activated T cells are still capable of producing a substantial quantity of cytokines [18]. Collectively, these findings suggested that CD4^+^ and CD8^+^ T cells were markedly activated and proliferated early after CTT, which was accompanied by the cell cycle and DNA replication arrest.

To further evaluate the changes in T cells at the early stage after CTT, the characteristics of splenic and peripheral CD4^+^ and CD8^+^ T cells on day 7 after CTT were analyzed by flow cytometry (Figure 1J). As shown in Figure 1K,L, the absolute number of splenic CD4^+^ T cells, and splenic and peripheral CD8^+^ T cells were significantly increased after CTT, indicating that CTT promoted T cell expansion. Additionally, in comparison to that in the tumor-bearing control group, the percentages of CD4^+^ Th1 cells in the CTT group were clearly elevated in the spleen and blood, and the percentage of splenic CD4^+^ Th2 cells was also increased (Figure 1M). Moreover, the CD4^+^ Th1 subset was the most prevalent of all the subsets in the spleen and blood after CTT (Figure 1M), suggesting that the differentiation of CD4^+^ T cells into Th1 cells was rapidly induced at the early stage after CTT. Moreover, the expression of IFN-γ in CD8^+^ T cells was markedly increased in the spleen and blood after CTT (Figure 1N). Meanwhile, the expression of perforin in peripheral CD8^+^ T cells showed an increasing tendency (Figure 1N). To further explore the cytotoxicity of CD8^+^ T cells on day 7 after CTT, splenic CD8^+^ T cells from the tumor-bearing control and CTT-treated mice were isolated via magnetic-activated cell sorting (MACS) and cocultured with calcein-AM-labeled B16F10 tumor cells to detect calcein fluorescence in the supernatant. Unexpectedly, the tumor killing capacity of splenic CD8^+^ T cells at the early stage after CTT was not significantly different from that of the CD8^+^ T cells from tumor-bearing controls (Figure S3). These results suggested that the cytotoxicity of splenic CD8^+^ T cells is not yet enhanced early after CTT. Overall, these findings demonstrated that CTT induces significant activation and proliferation of CD4^+^ and CD8^+^ T cells at the early stage, particularly promoting CD4^+^ Th1-dominant differentiation and the activation of CD8^+^ T cells.

### 3.2. Activated CD4^+^ and CD8^+^ T Cells Mediated Persistent Antitumor Immunity at the Early Stage After CTT

**Figure 2:**
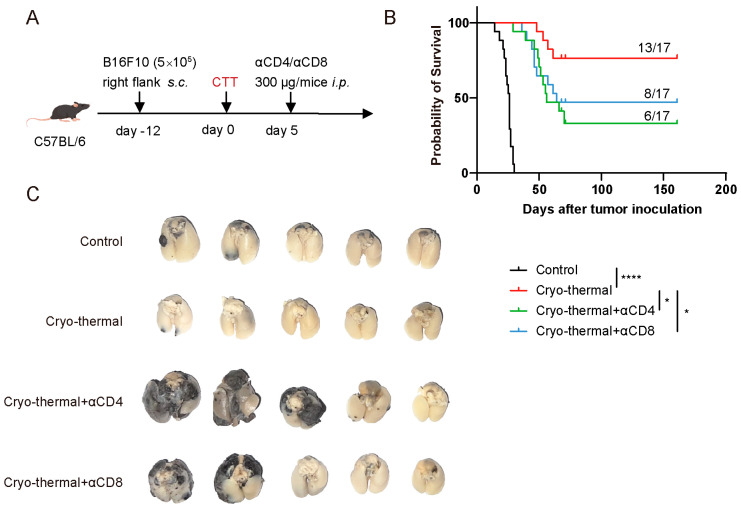
Activated CD4^+^ and CD8^+^ T cells at the early stage after CTT mediated long-term survival and prevented lung metastasis. (**A**) Scheme of the experiment design. A total of 300 μg of anti-CD4 or anti-CD8α antibodies were injected intraperitoneally on day 5 after CTT in the B16F10 model (n = 17). (**B**) Kaplan–Meier survival curve of the tumor-bearing control, CTT, CTT with CD4^+^ T cell depletion, and CTT with CD8^+^ T cell depletion groups. (**C**) The lung metastasis of each group (n = 5). * *p* < 0.05, **** *p* < 0.0001. In Kaplan–Meier plots, *p* values are determined via log-rank (Mantel–Cox) tests.

The above results demonstrated that CTT led to the rapid enhancement of the CD4^+^ Th1-dominant response and the activation of CD8^+^ T cells on day 7 after treatment. To determine whether the early activation of CD4^+^ and CD8^+^ T cells was crucial for inducing long-term antitumor immunity after CTT, 300 μg of anti-CD4 or anti-CD8α monoclonal antibodies were administered, *i.p.*, on day 5 after CTT to deplete CD4^+^ or CD8^+^ T cells, respectively (Figure 2A). Successful CD4^+^ and CD8^+^ T cell depletion within 14 days after injection was confirmed by flow cytometry (Figure S4A,B). Consistent with our previous studies, CTT significantly prolonged the survival time and improved the survival rate of B16F10 tumor-bearing mice (Figure 2B). Conversely, compared to that after CTT, depletion of CD4^+^ or CD8^+^ T cells resulted in a significantly lower survival rate, from 76.5% to 35.5% and 47%, respectively (Figure 2B). Moreover, CTT effectively prevented lung metastasis, whereas depletion of either CD4^+^ or CD8^+^ T cells led to a dramatic increase in lung metastases (Figure 2C), indicating that the loss of activated CD4^+^ or CD8^+^ T cells at the early stage after CTT compromised systemic antitumor immunity. These results highlighted that the CTT-induced activation of CD4^+^ and CD8^+^ T cells at the early stage is critical for establishing persistent antitumor immunity, as this activation effectively suppressed lung metastasis and promoted long-term survival in B16F10 tumor-bearing mice.

### 3.3. Crosstalk Occurred Between Activated CD4^+^ and CD8^+^ T Cells at the Early Stage After CTT

**Figure 3:**
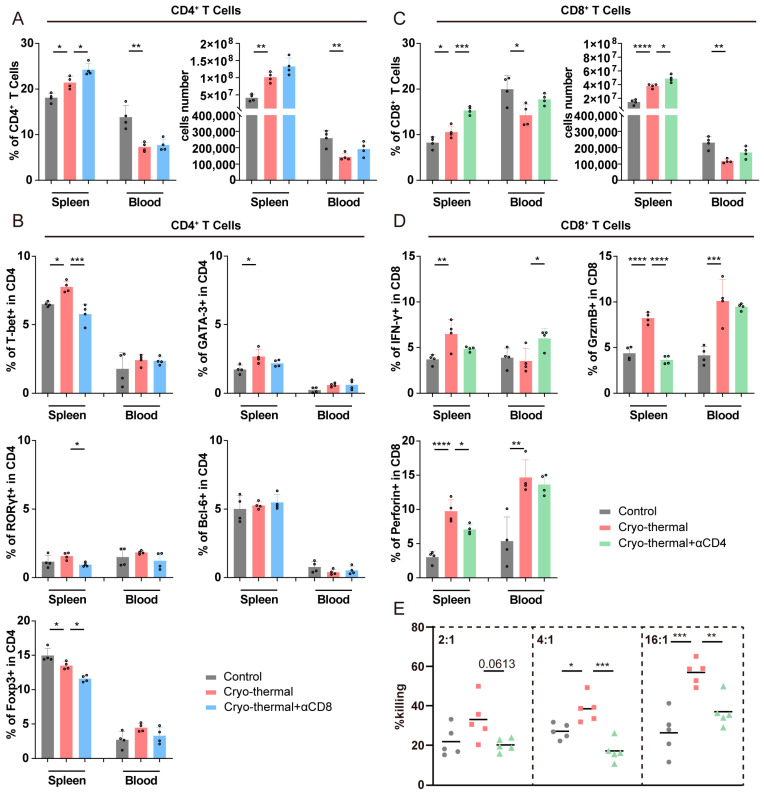
Crosstalk occurred between activated CD4^+^ and CD8^+^ T cells. (**A**) Proportion and absolute number of CD4^+^ T cells in the spleen and blood. (**B**) Subsets of CD4^+^ T cells in the spleen and blood. (**C**) Proportion and absolute number of CD8^+^ T cells in the spleen and blood. (**D**) IFN-γ, granzyme B, and perforin expression in CD8^+^ T cells in the spleen and blood. (**E**) Killing assay of splenic CD8^+^ T cells. All of the data are presented as the means ± SD. n = 4 for flow cytometry and n = 5 for killing assay. * *p* < 0.05, ** *p* < 0.01, *** *p* < 0.001, **** *p* < 0.0001. Data for graphs were calculated by one-way ANOVA.

To further investigate how activated T cells in the early phase after CTT affected long-term antitumor immunity in the B16F10 model, the T cell depletion strategy described above was employed on day 5 after CTT, and the changes in the immune cell population were subsequently analyzed on day 14 using flow cytometry. First, we investigated the changes in CD4^+^ and CD8^+^ T cells on day 14 after CTT with CD8^+^ or CD4^+^ T cell depletion at the early stage. As shown in Figure 3A, compared to those in the control group, the percentage and absolute number of splenic CD4^+^ T cells were increased after CTT, whereas reductions in the numbers of CD4^+^ T cells were observed in the blood. Additionally, the proportion of splenic CD4^+^ Th1 cells, as well as splenic CD4^+^ Th2 cells, was markedly increased after CTT, and CD4^+^ Th1 cells were the highest among all CD4^+^ T cell subsets (Figure 3B). Meanwhile, the proportion of CD4^+^ regulatory T cells (Tregs) was markedly decreased in the spleen (Figure 3B). However, after CD8^+^ T cell depletion, notable reductions in the numbers of Th1, Th17, and Treg cells in the spleen were noted compared to those in the CTT group (Figure 3B). Compared to those in the CTT group, other subsets of CD4^+^ T cells exhibited minimal changes in the depleted group. These results suggested that early-phase activated CD8^+^ T cells influence the differentiation of CD4^+^ T cells, particularly promoting CD4^+^ Th1-dominant differentiation at the late stage after CTT.

Furthermore, the percentage and absolute number of CD8^+^ T cells in the spleen were significantly increased on day 14 after CTT, whereas the proportion of these cells in the blood was decreased (Figure 3C). Additionally, after CTT, the expression of IFN-γ was significantly increased in the spleen, as well as the expression of granzyme B and perforin in splenic and peripheral CD8^+^ T cells (Figure 3D). Nevertheless, after CTT with CD4^+^ T cell depletion, there were significant increases in the proportion and absolute number of CD8^+^ T cells in the spleen, which was accompanied by notable reductions in the expression of granzyme B and perforin in CD8^+^ T cells in the spleen compared to those in the CTT group (Figure 3C,D). These findings indicated that the expression of cytotoxic molecules in CD8^+^ T cells in the late phase (day 14) was increased as a result of CD4^+^ T cell activation in the early phase (day 7). Furthermore, we investigated the killing capacity of splenic CD8^+^ T cells via a tumor killing assay. Compared to those from the tumor-bearing mice, CTT-induced CD8^+^ T cells exhibited increased cytotoxic activity toward B16F10 tumor cells in a proportion-dependent manner (Figure 3E). Nevertheless, the killing capacity of CD8^+^ T cells after CTT with CD4^+^ T cell depletion was significantly lower than that in the CTT group (Figure 3E). These findings indicated that CD4^+^ T cells at the early stage after CTT are essential for the cytotoxic functions of CD8^+^ T cells in the late phase.

Subsequently, alterations in innate immune cells were studied. Depletion of CD4^+^ or CD8^+^ T cells in the early phase had a limited effect on myeloid cells, including the population and maturation of G-MDSCs, M-MDSCs, DCs, and macrophages (Figure S5). Meanwhile, the population and cytotoxicity of NK cells remained minimally changed after depletion of T cells (Figure S6). These results suggested that activated T cells have a relatively small effect on the population and maturation of innate immune cells at the early stage after CTT.

In summary, these results indicated that there is a strong, mutual influence between activated CD4^+^ and CD8^+^ T cells during the early phase after CTT, which is crucial for the Th1-dominant differentiation of CD4^+^ T cells and CD8^+^ T cell cytotoxicity in the late phase after CTT.

### 3.4. The Mutual Influence of CTT-Activated CD4^+^ and CD8^+^ T Cells in the Early Phase Was Dependent on Cell–Cell Contact

**Figure 4:**
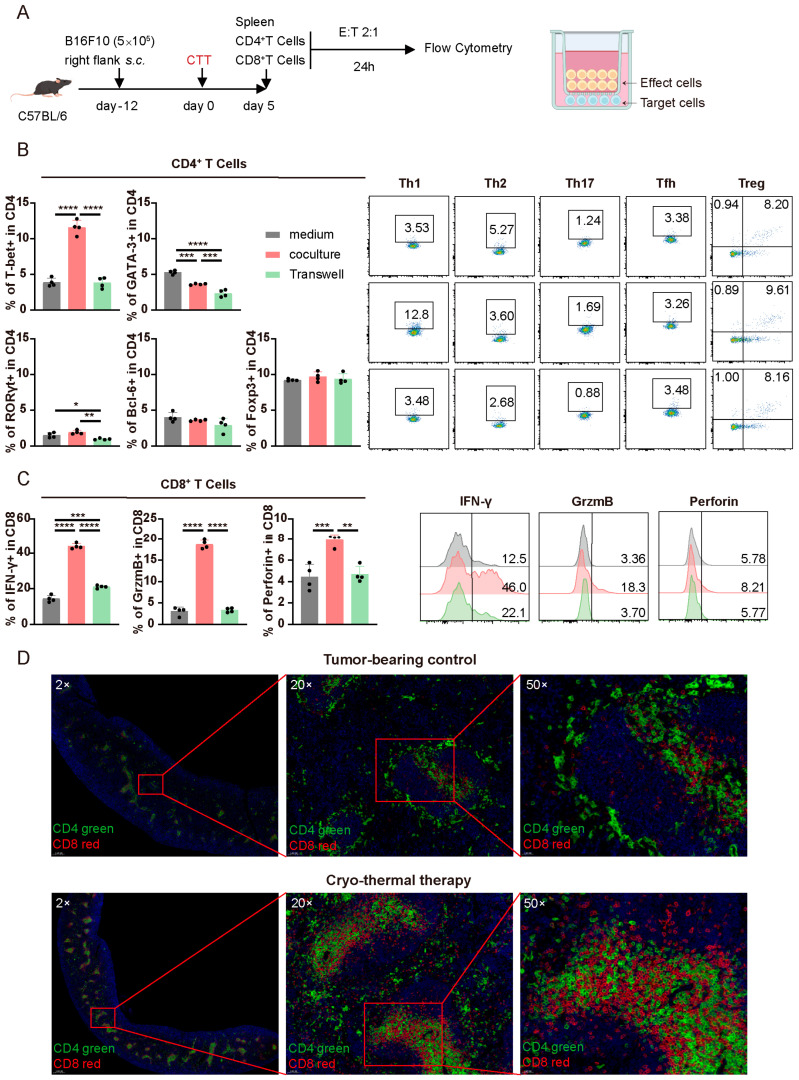
CTT-activated CD4^+^ and CD8^+^ T cells interacted via cell–cell contact. (**A**) Scheme of research design. CD4^+^ and CD8^+^ T cells were separately isolated by MACS on day 5 after CTT, and then cocultured at an effector-to-target ratio of 2:1 for 24 h. (**B**) Subsets of CD4^+^ T cells. (**C**) IFN-γ, granzyme B, and perforin expression in CD8^+^ T cells. (**D**) Immunofluorescence staining images of splenic CD4^+^ and CD8^+^ T cells of mice from the tumor-bearing or CTT group on day 7. All of the data are presented as the means ± SD. n = 4 for each group. * *p* < 0.05, ** *p* < 0.01, *** *p* < 0.001, **** *p* < 0.0001. Data for graphs were calculated by one-way ANOVA.

To validate the specific mechanism by which CD4^+^ and CD8^+^ T cells mutually influence each other, CD4^+^ and CD8^+^ T cells were isolated separately from the spleens of CTT-treated mice on day 5 and cocultured or separated in Transwell chambers for 24 h in vitro (Figure 4A). Compared to those cultured in medium, when CD4^+^ T cells were cocultured with CD8^+^ T cells, the proportion of CD4^+^ Th1 cells was markedly increased, the proportion of Th2 cells was slightly decreased, and the proportions of Th17, Tfh, and Treg cells remained unchanged (Figure 4B). Consistent with the in vivo results, CD4^+^ Th1 cells were present in the highest proportion among these subsets, indicating that activated CD8^+^ T cells predominantly promote the Th1-dominant differentiation of CD4^+^ T cells in the early phase after CTT. Nevertheless, the differentiation of CD4^+^ T cells into Th1, Th2, and Th17 cells was significantly diminished when CD4^+^ and CD8^+^ T cells were cultured separately in Transwell chambers (Figure 4B). These results suggested that activated CD8^+^ T cells in the early phase influence the Th1 differentiation of CD4^+^ T cells via cell–cell contact. Moreover, compared to those cultured in medium, CD8^+^ T cells cultured with CD4^+^ T cells presented significant increases in the expression levels of IFN-γ, granzyme B, and perforin (Figure 4C). Nevertheless, after CD8^+^ and CD4^+^ T cells were cultured separately in Transwell chambers, the increase in the expression of those cytotoxic molecules in CD8^+^ T cells was markedly diminished, indicating that activated CD4^+^ T cells affected the expression of cytotoxic molecules in CD8^+^ T cells via cell–cell contact in the early phase after CTT (Figure 4C). In conclusion, CD4^+^ and CD8^+^ T cells in the early phase after CTT have a strong mutual influence on facilitating the Th1-dominant CD4^+^ T cell differentiation and the expression of cytotoxic molecules in CD8^+^ T cells, which are mediated by direct cell–cell contact.

To further investigate the direct cell–cell contact between CD4^+^ and CD8^+^ T cells in vivo, immunofluorescence staining of splenic CD4^+^ and CD8^+^ T cells was conducted to evaluate their colocalization. As shown in Figure 4D, a greater degree of CD4^+^ and CD8^+^ T cell colocalization was observed in the CTT group than in the tumor-bearing group. These data demonstrated that CTT enhanced the aggregations of CD4^+^ and CD8^+^ T cells in vivo, which increasingly facilitated communication between these cells.

Overall, activated CD4^+^ and CD8^+^ T cells communicate directly through cell–cell contact to promote the differentiation of CD4^+^ T cells into Th1 cells and the expression of cytotoxic molecules in CD8^+^ T cells at the early stage after CTT.

### 3.5. CTT-Activated CD4^+^ and CD8^+^ T Cell Crosstalk Depended on the LFA-1/ICAM-1 Interaction

**Figure 5:**
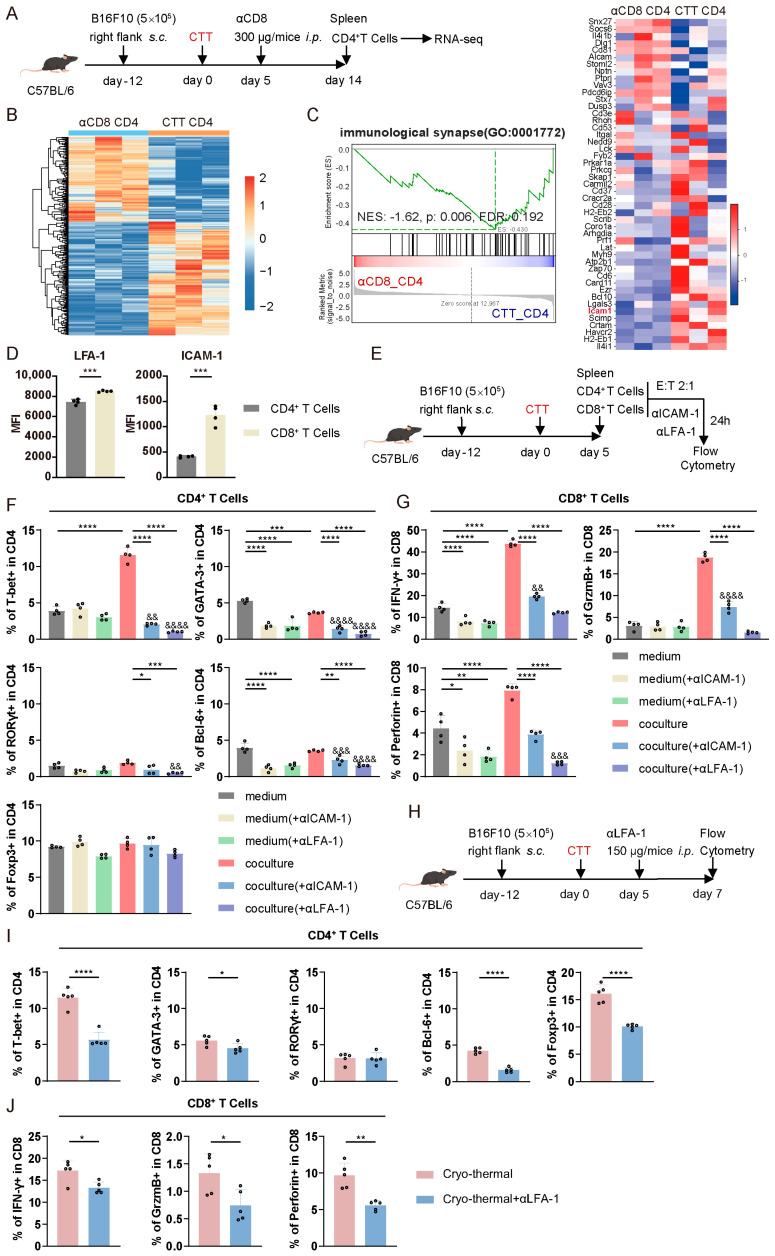
CTT-activated CD4^+^ and CD8^+^ T cells crosstalk depended on the LFA-1/ICAM-1 interaction. (**A**) Scheme of research design. A total of 300 μg of anti-CD8a antibodies were injected intraperitoneally on day 5 after CTT. All mice were sacrificed on day 14 to isolate splenic CD4^+^ T cells for RNA-seq. (**B**) Heatmap of differentially expressed genes in CD4^+^ T cells from the CTT group and the CD8^+^ T cell depletion group. (**C**) Gene enrichment of immunological synapse pathway according to GSEA in CD4^+^ T cells. (**D**) The expression of LFA-1 and ICAM-1 on CD4^+^ and CD8^+^ T cells. (**E**) Scheme of research design. CD4^+^ and CD8^+^ T cells were isolated by MACS on day 5 after CTT, and then cocultured at an effector-to-target ratio of 2:1 with the anti-ICAM-1 or LFA-1 antibodies for 24 h. (**F**) Subsets of CD4^+^ T cells. (**G**) IFN-γ, granzyme B, and perforin expression in CD8^+^ T cells. (**H**) Scheme of research design. A total of 150 μg of anti-LFA-1 antibodies were injected intraperitoneally on day 5 after CTT. All mice were sacrificed on day 7 to detect the phenotype of splenic CD4^+^ and CD8^+^ T cells. (**I**) Subsets of CD4^+^ T cells in the spleen. (**J**) IFN-γ, granzyme B, and perforin expression in CD8^+^ T cells in the spleen. All of the data are presented as the means ± SD. n = 3 for RNA-seq, n = 4 for in vitro experiment, n = 5 for in vivo experiment. * *p* < 0.05, ** *p* < 0.01, *** *p* < 0.001, **** *p* < 0.0001. Compared to the medium group, ^&&^ *p* < 0.01, ^&&&^ *p* < 0.001, ^&&&&^ *p* < 0.0001. Data for graphs (**D**) and (**I**,**J**) were calculated by Student’s *t*-test, and data for other graphs were calculated by one-way ANOVA.

To elucidate the underlying mechanism by which CD4^+^ Th1-dominant differentiation and CD8^+^ T cell activation are mediated via cell–cell contact after CTT, on day 14 after treatment, splenic CD4^+^ T cells from the CTT group and the CTT with CD8^+^ T cell depletion group were isolated and analyzed using RNA-seq (Figure 5A). A heatmap of the differentially expressed genes revealed that, compared to those from the CTT group, the CD4^+^ T cells from the CTT with anti-CD8α antibody treated group presented a distinct gene expression profile (Figure 5B). Moreover, GSEA revealed that cell–cell contact-related pathways, including the immunological synapse and cell adhesion molecules pathway in CD4^+^ T cells, were significantly downregulated after CTT with CD8^+^ T cell depletion (Figure 5C and Figure S7A–D). Then, the downregulated genes of the immunological synapse pathway were further investigated (Figure 5C). After excluding genes whose expression was low in CD4^+^ T cells, such as *Il4i1*, *H2-EB2*, *Scimp*, *Crtam,* and *Havcr2*, we focused on *ICAM-1*, which had high fragments per kilobase of transcript per million of mapped reads (FPKM). LFA-1 is the main receptor for ICAM-1, and the interaction between these two molecules promotes the T cell effector function and differentiation [19]. Accordingly, the expression of LFA-1 and ICAM-1 on T cells was observed by flow cytometry. LFA-1 was highly expressed on both CD4^+^ and CD8^+^ T cells, while ICAM-1 was highly expressed predominantly on CD8^+^ T cells (Figure 5D). Thus, we hypothesized that CD4^+^ Th1-dominant differentiation and enhancement of cytotoxic molecules expression in CD8^+^ T cells after CTT may be mediated by the LFA-1/ICAM-1 interaction. Therefore, splenic CD4^+^ and CD8^+^ T cells were isolated on day 5 after CTT and cocultured in vitro in the presence of anti-ICAM-1 or anti-LFA-1 antibodies for 24 h (Figure 5E). As shown in Figure 5F,G, the differentiation of the CD4^+^ Th1 subset and the expression levels of IFN-γ, granzyme B, and perforin in CD8^+^ T cells were significantly greater after the coculture of CD4^+^ and CD8^+^ T cells than in the cells cultured separately. However, after blocking ICAM-1 or LFA-1, the proportions of the Th1, Th2, Th17, and Tfh subsets among CD4^+^ T cells were significantly lower than the proportions of these subsets after coculturing with CD8^+^ T cells (Figure 5F). Moreover, anti-ICAM-1 or anti-LFA-1 antibodies abrogated the increase in the expression levels of IFN-γ, granzyme B, and perforin in CD8^+^ T cells after they were cocultured with CD4^+^ T cells, and the reduction in LFA-1 blocking was greater than that mediated by the blockade of ICAM-1 (Figure 5G). Conversely, blocking ICAM-1 or LFA-1 in CD4^+^ T cells cultured alone did not affect CD4^+^ Th1 differentiation, and only reduced the differentiation of CD4^+^ T cells into Th2 and Tfh cells (Figure 5F). Blocking LFA-1/ICAM-1 signaling in CD8^+^ T cells cultured alone resulted in a slight reduction in the expression of IFN-γ and perforin (Figure 5G). These findings indicated that LFA-1/ICAM-1 adhesion promotes CD4^+^ Th1-dominant differentiation and enhances CD8^+^ T cell-mediated cytotoxicity, which mainly exists in the interactions between CD4^+^ and CD8^+^ T cells, rather than within homotypic T cells.

The effect of the LFA-1/ICAM-1 interaction on T cells at the early stage after CTT was subsequently investigated in vivo. As the blockade of LFA-1 was more pronounced than the blockade of ICAM-1 during CD4^+^ and CD8^+^ T cell coculture (Figure 5G), anti-LFA-1 antibodies were injected intraperitoneally on day 5 after CTT (Figure 5H). Compared to those after CTT, the proportions of the Th1, Th2, Tfh, and Treg subsets after CTT with LFA-1 blocking were significantly reduced (Figure 5I). The most significant decrease was observed in the Th1 subset, indicating that LFA-1 signaling is essential for CTT-induced Th1-dominant differentiation (Figure 5I). Consistent with the in vitro results, LFA-1 blocking in vivo during the early phase after CTT resulted in a significant reduction in the expression of cytotoxic molecules, including IFN-γ, granzyme B, and perforin, in CD8^+^ T cells compared to CTT-induced CD8^+^ T cells (Figure 5J). These findings suggested that the LFA-1/ICAM-1 interaction is essential for CD4^+^ Th1-dominant differentiation and the expression of cytotoxic molecules in CD8^+^ T cells.

In summary, these results indicated that the LFA-1/ICAM-1-mediated interactions between activated CD4^+^ and CD8^+^ T cells facilitate CD4^+^ Th1-dominant differentiation and enhance the expression of cytotoxic molecules in CD8^+^ T cells.

### 3.6. The LFA-1/ICAM-1 Interaction Between CD4^+^ and CD8^+^ T Cells Promoted CD4^+^ Th1-Dominant Differentiation via the Notch1 Signaling Pathway and the Cytotoxic Function of CD8^+^ T Cells via the IL-2–STAT5 Signaling Pathway

**Figure 6:**
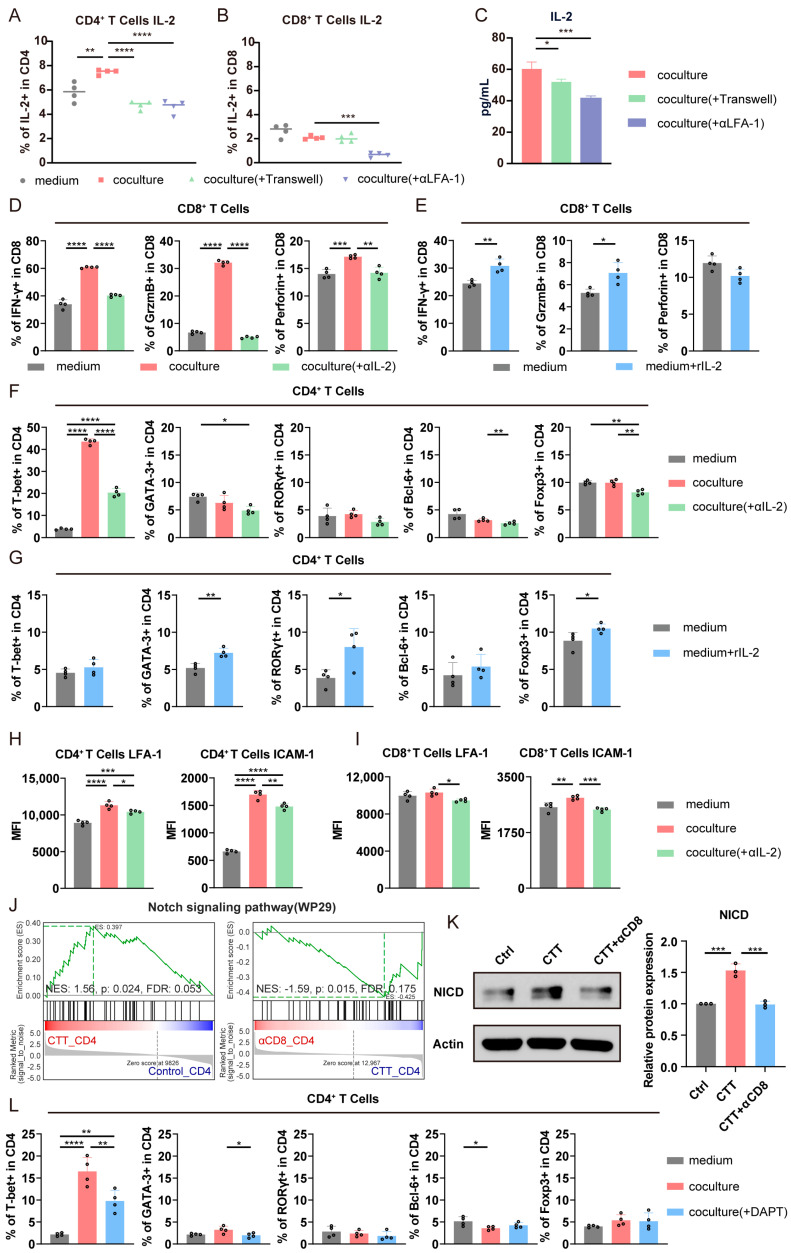
LFA-1/ICAM-1 interactions between CD4^+^ and CD8^+^ T cells promoted the expression of cytotoxic molecules in CD8^+^ T cells via IL-2-STAT5 signaling and the differentiation of CD4^+^ T cells into Th1 via Notch1 signaling. (**A**,**B**) The expression of IL-2 in (**A**) CD4^+^ and (**B**) CD8^+^ T cells. (**C**) The concentration of IL-2 in cultivation of supernatant. (**D**,**E**) IFN-γ, granzyme B, and perforin expression in CD8^+^ T cells (**D**) after IL-2 blockade or (**E**) rIL-2 supplementation. (**F**,**G**) Subsets of CD4^+^ T cells (**F**) after IL-2 blockade or (**G**) rIL-2 supplementation. (**H**,**I**) The mean fluorescence intensity of LFA-1 and ICAM-1 on (**H**) CD4^+^ and (**I**) CD8^+^ T cells. (**J**) The enrichment analysis of Notch signaling in CD4^+^ T cells. (**K**) The expression of NICD in CD4^+^ T cells. (**L**) Subsets of CD4^+^ T cells after blocking Notch1 signaling. All of the data are presented as the means ± SD. n = 4 for flow cytometry, n = 3 for RNA-seq or Western blot. * *p* < 0.05, ** *p* < 0.01, *** *p* < 0.001, **** *p* < 0.0001. Data for graphs (**E**) and (**G**) were calculated by Student’s *t*-test; data for other graphs were calculated by one-way ANOVA.

In the aforementioned experiment, we found that the IL-2–STAT5 signaling pathway in CD4^+^ and CD8^+^ T cells was significantly activated after CTT compared to that in tumor-bearing control mice (Figure 1G,I). Moreover, the LFA-1-dependent cell–cell adhesion of CD8^+^ T cells promoted the production of IL-2 [20]. Thus, we hypothesized that LFA-1/ICAM-1 signaling would induce the production of IL-2 to promote the expression of cytotoxic molecules in CD8^+^ T cells and CD4^+^ Th1-dominant differentiation. As shown in Figure 6A, the expression of IL-2 in CD4^+^ T cells was significantly increased when CD4^+^ T cells were cocultured with CD8^+^ T cells than when they were cultured in medium alone. Concurrently, the expression of IL-2 in CD4^+^ T cells was significantly reduced when CD4^+^ T cells were separately cultured in Transwell chambers or treated with the anti-LFA-1 antibody (Figure 6A). In addition, the expression of IL-2 in CD8^+^ T cells was much lower than that in CD4^+^ T cells, and was not obviously different after CD8^+^ T cells were cocultured with CD4^+^ T cells compared to those cultured in medium alone (Figure 6B). Following this, the concentration of IL-2 in the cell culture supernatant was subsequently measured by an enzyme-linked immunosorbent assay (ELISA). The IL-2 concentration in the supernatant was significantly decreased when the T cells were separately cultured in Transwell chambers or treated with the anti-LFA-1 antibody than when they were cocultured (Figure 6C). These results indicated that the interaction between CD4^+^ and CD8^+^ T cells via LFA-1/ICAM-1 promotes the secretion of IL-2 in CD4^+^ T cells.

Next, we explored whether IL-2 production induced via the LFA-1/ICAM-1 interaction affects the expression of cytotoxic molecules in CD8^+^ T cells and the differentiation of CD4^+^ T cells into Th1 cells. Consistent with the previous results, the expression of IFN-γ, granzyme B, and perforin was significantly increased in CD8^+^ T cells after coculturing with CD4^+^ T cells (Figure 6D and Figure S8A). However, this increase was abolished by treatment with the IL-2 neutralizing antibody or the STAT5 inhibitor BD750 (Figure 6D and Figure S8A). Moreover, supplementation with rIL-2 directly resulted in an elevated expression of IFN-γ and granzyme B in CD8^+^ T cells (Figure 6E). The above findings indicated that CD4^+^ T cell-secreted IL-2 induced by the LFA-/ICAM-1 interaction promoted the expression of cytotoxic molecules in CD8^+^ T cells.

Similarly, there were more CD4^+^ T cells differentiated into Th1 cells when CD4^+^ T cells were cocultured with CD8^+^ T cells, but this increase was abolished by treatment with the IL-2 neutralizing antibody or BD750 (Figure 6F and Figure S8B). Meanwhile, administration of the IL-2 neutralizing antibody or BD750 only slightly decreased the proportions of other CD4^+^ T cell subsets (Figure 6F and Figure S8B). Surprisingly, when CD4^+^ T cells were cultured in medium, the addition of rIL-2 did not promote CD4^+^ Th1-dominant differentiation; instead, the differentiation of CD4^+^ T cells into Th2, Th17, and Treg cells was increased (Figure 6G), which suggested that the premise of IL-2 promoting the differentiation of CD4^+^ T cells into Th1 cells is their interaction with CD8^+^ T cells. Furthermore, IL-2–STAT5 signaling can regulate the expression of LFA-1 on CD8^+^ T cells [21]. Therefore, we hypothesized that IL-2 promotes CD4^+^ Th1-dominant differentiation by upregulating the expression of LFA-1 or ICAM-1 on T cells, thereby enhancing LFA-1/ICAM-1 adhesion between CD4^+^ and CD8^+^ T cells. Consequently, we evaluated the effects of IL-2 on the expression of LFA-1 and ICAM-1 on T cells using flow cytometry. Compared to the cells cultured in medium alone, the expression of LFA-1 and ICAM-1 on CD4^+^ T cells and the expression of ICAM-1 on CD8^+^ T cells was increased after coculturing, whereas the expression of these molecules was decreased in the presence of the IL-2 neutralizing antibody or BD750 (Figure 6H,I and Figure S8C,D). The results demonstrated that a high level of IL-2, which is mainly secreted by CD4^+^ T cells via CD4^+^ and CD8^+^ T cell interactions, increase the intensity of LFA-1/ICAM-1 signaling, leading to further promotion of the differentiation of CD4^+^ T cells into Th1 cells.

Thus, we aimed to investigate how LFA-1/ICAM-1 signaling affects CD4^+^ Th1 differentiation. The RNA-seq results indicated that Notch signaling in CD4^+^ T cells was significantly activated after CTT, whereas it was significantly suppressed after CD8^+^ T cell depletion (Figure 6J). Notch1 signaling requires the release of the Notch1 intracellular domain (NICD) from the membrane to facilitate its nuclear translocation for transcriptional activation [22]. Therefore, we analyzed NICD expression in splenic CD4^+^ T cells from different groups via Western blotting. The results demonstrated that CTT triggered significant upregulation of NICD in CD4^+^ T cells compared to that in tumor-bearing controls, which was abrogated by CD8^+^ T cell depletion after CTT (Figure 6K), indicating that CD8^+^ T cells are indispensable for CTT-induced Notch1 activation in CD4^+^ T cells. Furthermore, given the important role of Notch1 signaling in CD4^+^ Th1 differentiation [23], we added the inhibitor of Notch1 signaling, DAPT, to the coculture system to investigate its effect on CD4^+^ T cell differentiation. We found that inhibiting Notch1 signaling in the CD4^+^ and CD8^+^ T cell coculture system significantly reduced the differentiation of CD4^+^ T cells into Th1 cells and slightly inhibited the differentiation of CD4^+^ T cells into Th2 cells (Figure 6L). These results suggested that CD8^+^ T cells promote CD4^+^ Th1-dominant differentiation by Notch1 signaling, which is triggered by the LFA-1/ICAM-1 interaction between CD8^+^ and CD4^+^ T cells.

The above flow cytometry and immunofluorescence results of day 7 after CTT demonstrated that CTT significantly increased the LFA-1/ICAM-1 interaction between CD4^+^ and CD8^+^ T cells, promoting CD4^+^ Th1-dominant differentiation and the expression of cytotoxic molecules in CD8^+^ T cells. Moreover, when T cells were cultured in vitro, we found that the number of live cells was increased in the CD4^+^ and CD8^+^ T cell coculture system, whereas blocking LFA-1 decreased the number of live cells (Figure S9A). Furthermore, IL-2 plays crucial roles in T cell survival and proliferation [24]. Thus, we hypothesized that IL-2 induced by the LFA-1/ICAM-1 interaction between CD4^+^ and CD8^+^ T cells would increase the viability and proliferation of T cells. Annexin V/PI and Ki67 staining of T cells, followed by flow cytometry analysis, revealed significant increases in CD4^+^ and CD8^+^ T cell viability and proliferation after coculturing compared to those after culture alone (Figure S9B–E). However, the viability of CD4^+^ and CD8^+^ T cells and their expression of Ki67 were notably reduced when they were treated with LFA-1 blocking antibodies or IL-2 neutralizing antibodies than when CD4^+^ and CD8^+^ T cells were cocultured (Figure S9B–E). These results indicated that the LFA-1/ICAM-1 interaction between CD4^+^ and CD8^+^ T cells promote their viability and proliferation via IL-2.

To validate the generalizability of the CD4^+^ and CD8^+^ T cell interaction mechanism after CTT, further experiments were performed in an in vitro 4T1 tumor model (Figure S10A). The results revealed that the expression of cytotoxic molecules in CD8^+^ T cells and the differentiation of CD4^+^ T cells into Th1 cells were significantly increased after coculturing than those cultured in medium alone (Figure S10B,C). Conversely, the increases in the expression of cytotoxic molecules in CD8^+^ T cells and CD4^+^ Th1-dominant differentiation were abolished when the cells were separately cultured in Transwell chambers or treated with LFA-1 blocking or IL-2 neutralizing antibodies (Figure S10B,C). These results demonstrated that the interaction mechanism between CTT-activated CD4^+^ and CD8^+^ T cells exists in 4T1 tumor models.

In summary, the results demonstrated that CTT promotes the interaction between CD4^+^ and CD8^+^ T cells via LFA-1/ICAM-1 binding, in which CD8^+^ T cells trigger Notch1 signaling in CD4^+^ T cells to induce Th1-dominant differentiation and increase the production of IL-2 in CD4^+^ T cells. IL-2 produced by CD4^+^ T cells further promotes the expression of LFA-1 and ICAM-1 on T cells, forming a positive feedback loop that enhances the interaction between CD4^+^ and CD8^+^ T cells. In addition, the IL-2 secreted by CD4^+^ T cells directly induces the expression of cytotoxic molecules in CD8^+^ T cells.

### 3.7. LFA-1/ICAM-Dependent Interactions Between CD4^+^ and CD8^+^ T Cells Were Correlated with Clinical Outcomes

**Figure 7:**
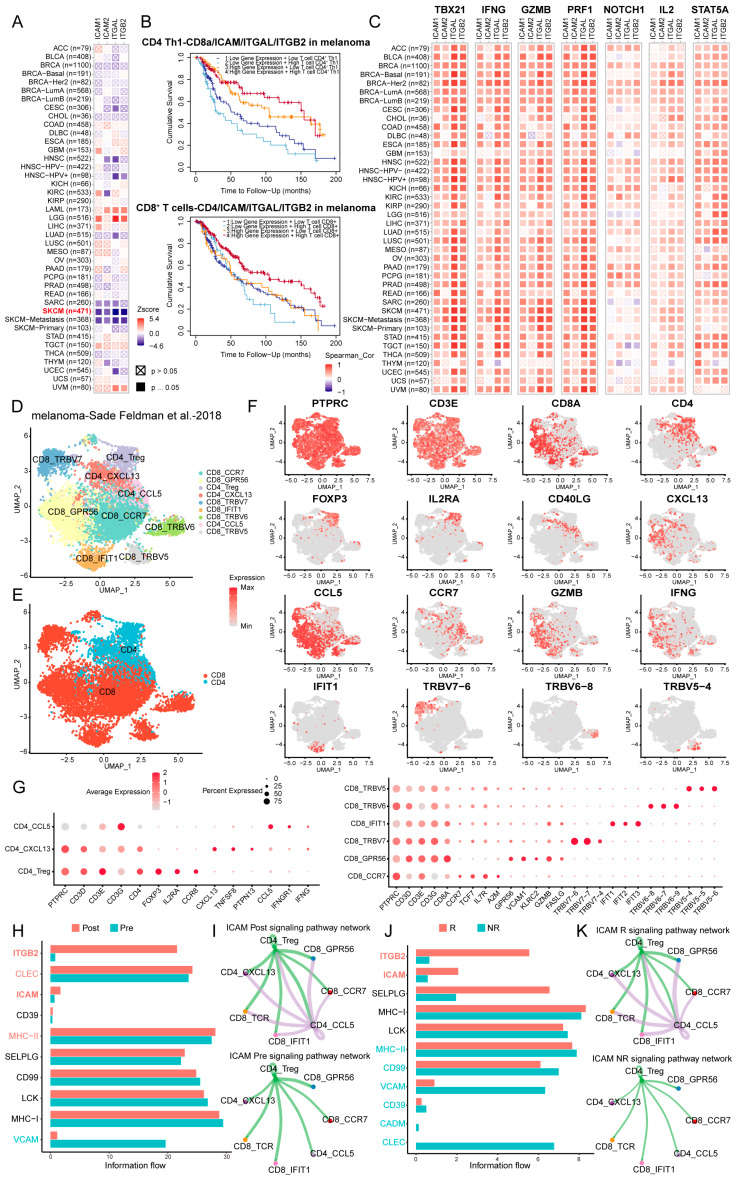
Clinical outcomes associated with LFA-1/ICAM-dependent interaction between CD4^+^ and CD8^+^ T cells. (**A**) Heatmap showing the associations between clinical outcomes and the expression of *ICAM, ITGAL,* and *ITGB2* across multiple cancer types from TIMER 2.0 database. (**B**) The Kaplan–Meier survival curve of the expression of *ICAM, ITGAL, ITGB2,* and T cell infiltration in predicting melanoma survival from TIMER 2.0 database. (**C**) Heatmap showing the associations between *ICAM, ITGAL,* and *ITGB2* with T cell activation markers and the involved in mechanism markers. (**D**) UMAP of the 9 clusters of the T cell. (**E**) CD4^+^ T cells and CD8^+^ T cells were identified by marker genes. (**F**) Feature plot of the different marker gene expressions across CD4^+^ and CD8^+^ T cells. (**G**) Dot plot of the different marker genes of CD4^+^ T cells (left) and CD8^+^ T cells (right). (**H**) The communication probability of pathways and (**I**) the *ICAM* signaling pathway net between CD4^+^ and CD8^+^ T cells from pre-treatment to post-treatment, analyzed by CellChat. (**J**) The communication probability of pathways and (**K**) the *ICAM* signaling pathway net between CD4^+^ and CD8^+^ T cells from nonresponders to responders, analyzed by CellChat [17].

To evaluate the clinical relevance of LFA-1/ICAM-mediated CD4^+^ and CD8^+^ T cell interactions, we comprehensively examined the clinical and genomic features of tumors using TIMER 2.0. Our analysis revealed that a higher expression of *ICAM, ITGAL (LFA-1α),* and *ITGB2 (LFA-1β)* was significantly correlated with a low risk for most cancer types, particularly melanoma (Figure 7A). Patients exhibiting concurrent high infiltration of CD4^+^ Th1 and CD8^+^ T cells and elevated *ICAM, ITGAL,* and *ITGB2* expression had the best clinical outcomes out of the patients with melanoma (Figure 7B). Moreover, the expression of these genes was positively correlated with the expression of T cell activation markers (*TBX21, IFNG, GZMB,* and *PRF1*) and signaling molecules involved in LFA-1/ICAM-mediated T cell interactions (*NOTCH1, IL2,* and *STAT5A*) (Figure 7C). These results indicated that the LFA-1/ICAM axis is linked to improved clinical outcomes and enhanced T cell immune responses.

We further analyzed scRNA-seq data from 48 biopsied melanoma tissues (32 patients in immune checkpoint blockade (ICB) clinical trials, acquired from the 3CA database [17]), including 19 pretreatment samples (nonresponders (NR), n = 10; and responders (R), n = 9) and 29 post-treatment samples (NR, n = 21, and R, n = 8) with 16,291 high-quality CD45^+^ cells. After normalization and principal component analysis (PCA) were performed, a total of 8915 T cells were clustered into nine subsets, including three clusters of CD4^+^ T cells and six clusters of CD8^+^ T cells (Figure 7D,E). Furthermore, we employed specific markers to annotate the nine clusters, comprising CD4^+^ CCL5^+^ cells (*CCL5, IFNGR1,* and *IFNG*), CD4^+^ CXCL13^+^ cells (*CXCL13, TNFSF8,* and *PTPN13*), CD4^+^ Tregs (*FOXP3, IL2RA,* and *CCR8*), CD8^+^ GPR56^+^ cells (*GPR56, VCAM1, KLRC2, GZMB,* and *FASLG*), CD8^+^ IFIT1^+^ cells (*IFIT1, IFIT2,* and *IFIT3*), CD8^+^ CCR7^+^ cells (*CCR7, TCF7, IL7R,* and *A2M*), CD8^+^ TRBV5^+^ cells (*TRBV5-4, TRBV5-5,* and *TRBV5-6*), CD8^+^ TRBV6^+^ cells (*TRBV6-7, TRBV6-8,* and *TRBV6-9*), and CD8^+^ TRBV7^+^ cells (*TRBV7-4, TRBV7-6,* and *TRBV7-7*) (Figure 7F,G). A cell–cell interaction analysis revealed that *ITGB2* and *ICAM*-mediated interactions between CD4^+^ and CD8^+^ T cells had marked increases in the post-treatment samples compared to the pre-treatment samples (Figure 7H). Furthermore, the *ICAM*-mediated interactions between CD4^+^ CCL5^+^ cells and all CD8^+^ T cell clusters were enhanced after ICB treatment (Figure 7I). These results demonstrated that the LFA-1/ICAM interactions between CD4^+^ and CD8^+^ T cells were increased in patients receiving ICB therapy. Moreover, compared to nonresponders, responders presented higher *ITGB2* and *ICAM* signaling between CD4^+^ and CD8^+^ T cells before treatment (Figure 7J). Visualization of the *ICAM* interaction network highlighted the significantly stronger connectivity between CD4^+^ CCL5^+^ cells and all CD8^+^ T cell clusters in responders than in nonresponders (Figure 7K). These results revealed that higher baseline levels of LFA-1/ICAM-dependent CD4^+^ and CD8^+^ T cell interactions were associated with greater responsiveness to ICB. Overall, these results suggest that the LFA-1/ICAM-dependent T cell interactions are correlated with a favorable prognosis, and could serve as a potential biomarker to predict responsiveness to ICB therapy.

## 4. Discussion

In this study, CTT-activated CD4^+^ and CD8^+^ T cells were indicated to be essential for the long-term survival of mice. We found that the aggregations of and interactions between activated CD4^+^ and CD8^+^ T cells were enhanced after CTT, in which CD8^+^ T cells promoted the differentiation of CD4^+^ T cells into Th1 cells by activating Notch1 signaling in CD4^+^ T cells, and CD4^+^ T cells enhanced the cytotoxicity of CD8^+^ T cells by secreting IL-2, which was attributed to LFA-1/ICAM-1 adhesion. An analysis of clinical databases also revealed that increased LFA-1/ICAM-mediated interactions between CD4^+^ and CD8^+^ T cells were correlated with favorable clinical outcomes in patients with melanoma. This study revealed the novel mechanism of the LFA-1/ICAM-1 adhesion pathway between CD4^+^ and CD8^+^ T cell interactions for T cell activation after CTT.

The helper functions of CD4^+^ T cells in the activation of CD8^+^ T cells for antitumor immunity are well-understood. CD4^+^ Th1 cell-derived IFN-γ promotes the survival, proliferation, and maturation of CD8^+^ T cells [25,26] and directly enhances cytotoxic CD8^+^ T cell differentiation [16]. Furthermore, CD4^+^ Th1 cells potentiate CD8^+^ T cell responses by producing IL-2, by which the expression of Blimp-1, Id2, and T-bet in CD8^+^ T cells is induced to exhibit robust proliferation, as well as the transcription of *Eomes* and *Prf1* in CD8^+^ T cells, which is increased to facilitate their effector functions [27,28]. A recent study has highlighted that CD4^+^ T cells and the intratumoral immune triad are necessary for the cytotoxicity and tumor elimination of CD8^+^ T cells at the effector-stage [7]. However, few studies have focused on how CD8^+^ T cells regulate CD4^+^ T cells, especially the role of CD8^+^ T cells in the differentiation of CD4^+^ Th1 cells. It has only been found that in the presence of CD8^+^ T cells, stimulation of healthy CD4^+^ T cells could induce more effector CD4^+^ T cells and more homogeneous CD25 expression through cytokine and Fas/FasL signaling [29]. In the HIV-infected model, CD8^+^ T cells affect the metabolic and transcriptional states of memory CD4^+^ T cells to induce HIV latency [30]. In this study, we found that CTT-activated CD8^+^ T cells interact with CD4^+^ T cells via LFA-1/ICAM-1. Activation of LFA-1/ICAM-1 signaling on CD4^+^ T cells triggered Notch1 signaling to promote Th1 differentiation, which is consistent with the findings reported by Vrema et al. [31]. Furthermore, the interactions between CD4^+^ and CD8^+^ T cells amplify the secretion of IL-2 from CD4^+^ T cells, which subsequently activates STAT5 signaling to potentiate the cytotoxic effector functions of CD8^+^ T cells. Meanwhile, IL-2 secreted by CD4^+^ T cells established a positive feedback loop to further enhance the T cell activation via promoting the expression of LFA-1 and ICAM-1 on both T cells. Collectively, our findings elucidate a previously unrecognized mechanism by which CD8^+^ T cells orchestrate CD4^+^ T cell differentiation through the LFA-1/ICAM-1 axis and show that heterotypic T cell communication promotes the differentiation of CD4^+^ T cells into Th1 cells and the cytotoxicity of CD8^+^ T cells, thereby sustaining long-term CTT-induced antitumor immunity.

LFA-1 and ICAM-1 are adhesion molecules critical for immune synapse formation and T cell activation [32]. The LFA-1/ICAM-1 axis orchestrates T cell–antigen-presenting cell (APC) synaptic stabilization, whereas mechanical signaling through LFA-1 reinforces pMHC engagement to amplify antigen-dependent T cell activation [33]. In addition, the interaction between LFA-1 and ICAM-1 within homotypic T cells has been identified as a pivotal factor for their activation and differentiation [34]. Moreover, this LFA-1/ICAM-1-mediated homotypic T cell crosstalk results in the formation of multifocal synapses that promote the targeted delivery of cytokines, including IL-2 and IFN-γ, which also have costimulatory properties for T cell activation [35,36,37]. In this study, we found that LFA-1/ICAM-1 adhesion between homotypic CD8^+^ T cells has a relatively small effect on the enhancement of CD8^+^ T cell cytotoxicity, and that this adhesion between homotypic CD4^+^ T cells fails to drive CD4^+^ Th1-dominant differentiation. Strikingly, LFA-1/ICAM-1 interactions between CD4^+^ and CD8^+^ T cells markedly enhanced CD4^+^ Th1-dominant differentiation and the cytotoxic functions of CD8^+^ T cells. To investigate the causes of the enhanced interactions between CD4^+^ and CD8^+^ T cells, we found that CTT induced the expansion of T cells (Figure 1K,L), which induced pronounced aggregations of CD4^+^ and CD8^+^ T cells (Figure 4D). Moreover, the expression of ICAM-1 on CD8^+^ T cells was greater than that on CD4^+^ T cells (Figure 5D); thus, a strong LFA-1/ICAM-1 interaction occurred between CD4^+^ and CD8^+^ T cells, which triggered Notch1 signaling in CD4^+^ T cells for their differentiation into Th1 cells, and facilitated their secretion of IL-2. Although LFA-1/ICAM-1 interactions between homotypic CD8^+^ T cells led to a slight increase in the expression of cytotoxic molecules in CD8^+^ T cells, CD4^+^ T cell-derived IL-2 significantly enhanced the cytotoxic functions of CD8^+^ T cells. However, the present study did not provide direct evidence that ICAM-1 on CD8^+^ T cells provides the initial signal for the differentiation of CD4^+^ Th1 cells. Consequently, the future studies will employ ICAM-1-deficient CD8^+^ T cells cultured with WT CD4^+^ T cells to observe the differentiation of CD4^+^ T cells, which will present important evidence for the comprehensive mechanism of the LFA-1/ICAM-1 interaction between CD4^+^ and CD8^+^ T cells.

Recent evidence has shown that CD4^+^ and CD8^+^ T cell infiltration is associated with improved prognosis across multiple cancer types [38,39,40]. Consistently, our analysis of clinical data revealed that LFA-1/ICAM-mediated interactions between CD4^+^ and CD8^+^ T cells were significantly increased after ICB and elevated T cell activation signatures. Moreover, patients exhibiting pre-treatment enrichment of LFA-1/ICAM-mediated T cell interactions displayed greater responsiveness to ICB, which suggests that these interactions may potentiate the efficacy of ICB and serve as potential biomarkers to predict ICB responsiveness. Overall, these findings underscore the necessity of incorporating CD4^+^ and CD8^+^ T cell interaction dynamics into clinical evaluations to further expand our understanding of the mechanisms underlying the favorable clinical efficacy of CTT.

Our previous studies revealed that CTT reprogrammed the immunosuppressive environment in the early stage, which lead to the systemic activation and maturation of multiple immune cells and ultimately induced CD4^+^ Th1-dominant differentiation after CTT [16]. CTT-induced M1 macrophages and mature DCs can directly promote the differentiation of CD4^+^ T cells toward Th1 cells [15]. Concurrently, NK cells activated during the early stage after CTT can reprogram MDSCs toward a mature phenotype, which, in turn, orchestrates CD4^+^ Th1-dominant differentiation by enhancing antigen presentation [41]. In this study, we identified a novel mechanism in which CD8^+^ T cells activate Notch1 signaling in CD4^+^ T cells via the LFA-1/ICAM-1 interaction to promote the differentiation of CD4^+^ T cells into Th1 cells. Overall, our studies revealed the coordinated interactions among various immune cells in the early stage after CTT to promote the Th1-dominant differentiation of CD4^+^ T cells, ultimately sustaining long-term antitumor immune memory after CTT.

In order to improve the antitumor immunity efficacy of CTT in malignant tumors, combined therapies may be the future of oncology. A recent study has shown that an immune stimulator of LFA-1 and VLA-1, 7HP349, is used in combination with CTLA-4 inhibitors to improve the elimination of tumors [42]. In this study, we found the LFA-1/ICAM-1 interactions between CD4^+^ and CD8^+^ T cells could promote T cell activation and effective functions. Future studies should systematically evaluate whether there is therapeutic potential of augmented LFA-1/ICAM-1 interactions in enhancing CTT-mediated antitumor immune responses in murine tumor models, which could provide crucial mechanistic insights for optimizing CTT-based immunotherapy protocols and broadening their clinical applicability.

## 5. Conclusions

In conclusion, our study revealed that activated CD4^+^ and CD8^+^ T cells interacted via LFA-1/ICAM-1 after CTT, in which CD8^+^ T cells facilitated Notch1 signaling in CD4^+^ T cells to promote CD4^+^ Th1-dominant differentiation, and the IL-2 secreted from CD4^+^ T cells enhanced the cytotoxic functions of CD8^+^ T cells. Moreover, IL-2 secreted from CD4^+^ T cells increased the expression of LFA-1 and ICAM-1 on T cells to establish a positive feedback loop. Our findings revealed a novel mechanism of CD4^+^ and CD8^+^ T cell interaction after CTT that contributes to sustaining antitumor immunity.

## Data Availability

The data presented in this study are available upon request from the corresponding author.

## Supplementary Materials

The following supporting information can be downloaded at: https://www.mdpi.com/article/10.3390/cells14080620/s1, Figure S1: Gating strategy of flow cytometry; Figure S2: GSEA of CD4^+^ and CD8^+^ T cells in the tumor-bearing control and CTT groups; Figure S3: The tumor killing capacity of CD8^+^ T cells from tumor-bearing control and CTT-treated mice; Figure S4: The percentage of CD4^+^ and CD8^+^ T cells after T cell depletion; Figure S5: The amount and phenotype of myeloid cells under depletion of T cells after CTT in vivo; Figure S6: The amount and phenotype of NK cells under depletion of T cells after CTT in vivo; Figure S7: GSEA of CD4^+^ T cells in the CTT and CTT with CD8^+^ T cell depletion groups; Figure S8: The role of STAT5 in CD4^+^ and CD8^+^ T cells interaction; Figure S9: The LFA-1/ICAM-1-IL-2 axis between CD4^+^ and CD8^+^ T cells promote the viability and proliferation of T cells; Figure S10: The phenotype of CD4^+^ and CD8^+^ T cells in 4T1 tumor model; Table S1: Antibodies used for flow cytometry.

## Abbreviations

The following abbreviations are used in this manuscript:

CTT	cryo-thermal therapy
Th1	T helper type 1
Th2	T helper type 2
Th17	T helper type 17
Tfh	T follicular helper
Treg	regulatory T cell
GrzmB	granzyme B
CTL	Cytotoxic T lymphocytes
APC	antigen-presenting cell
MDSC	myeloid-derived suppressor cell
DC	dendritic cell
MHC II	major histocompatibility complex class II
DAMP	damage-associated molecular pattern
IFN	interferon
IL	interleukin
LFA-1	leukocyte function-associated antigen
ICAM-1	intercellular adhesion molecule 1
NICD	Notch1 intracellular domain
ICB	immune checkpoint blockade
DMEM	Dulbecco’s modified eagle’s medium
FBS	fetal bovine serum
ELISA	enzyme-linked immunosorbent assay
MACS	magnetic-activated cell sorting
GSEA	gene set enrichment analysis
PVDF	polyvinylidene fluoride
i.p	intraperitoneal injection
s.c	subcutaneous injection
SD	standard deviation

## References

[1] Sun L., Su Y., Jiao A., Wang X., Zhang B. (2023). T cells in health and disease. Signal Transduct. Target. Ther..

[2] Raskov H., Orhan A., Christensen J.P., Gögenur I. (2021). Cytotoxic CD8+ T cells in cancer and cancer immunotherapy. Br. J. Cancer..

[3] Chen Y., Yu D., Qian H., Shi Y., Tao Z. (2024). CD8(+) T cell-based cancer immunotherapy. J. Transl. Med..

[4] Speiser D.E., Chijioke O., Schaeuble K., Münz C. (2023). CD4(+) T cells in cancer. Nat. Cancer.

[5] Kravtsov D.S., Erbe A.K., Sondel P.M., Rakhmilevich A.L. (2022). Roles of CD4+ T cells as mediators of antitumor immunity. Front. Immunol..

[6] Ferris S.T., Durai V., Wu R., Theisen D.J., Ward J.P., Bern M.D., Davidson J.T., Bagadia P., Liu T., Briseño C.G. (2020). cDC1 prime and are licensed by CD4(+) T cells to induce anti-tumour immunity. Nature.

[7] Espinosa-Carrasco G., Chiu E., Scrivo A., Zumbo P., Dave A., Betel D., Kang S.W., Jang H.-J., Hellmann M.D., Burt B.M. (2024). Intratumoral immune triads are required for immunotherapy-mediated elimination of solid tumors. Cancer Cell.

[8] Magen A., Hamon P., Fiaschi N., Soong B.Y., Park M.D., Mattiuz R., Humblin E., Troncoso L., D’souza D., Dawson T. (2023). Intratumoral dendritic cell-CD4(+) T helper cell niches enable CD8(+) T cell differentiation following PD-1 blockade in hepatocellular carcinoma. Nat. Med..

[9] Borsetto D., Tomasoni M., Payne K., Polesel J., Deganello A., Bossi P., Tysome J.R., Masterson L., Tirelli G., Tofanelli M. (2021). Prognostic Significance of CD4+ and CD8+ Tumor-Infiltrating Lymphocytes in Head and Neck Squamous Cell Carcinoma: A Meta-Analysis. Cancers.

[10] Liu D., Heij L.R., Czigany Z., Dahl E., Lang S.A., Ulmer T.F., Luedde T., Neumann U.P., Bednarsch J. (2022). The role of tumor-infiltrating lymphocytes in cholangiocarcinoma. J. Exp. Clin. Cancer Res. CR.

[11] Fu Q., Chen N., Ge C., Li R., Li Z., Zeng B., Li C., Wang Y., Xue Y., Song X. (2019). Prognostic value of tumor-infiltrating lymphocytes in melanoma: A systematic review and meta-analysis. Oncoimmunology.

[12] Xie Y., Liu P., Xu L.X. A novel thermal treatment modality for controlling breast tumor growth and progression. Proceedings of the Annual International Conference of the IEEE Engineering in Medicine and Biology Society IEEE Engineering in Medicine and Biology Society Annual International Conference.

[13] Zhu J., Zhang Y., Zhang A., He K., Liu P., Xu L.X. (2016). Cryo-thermal therapy elicits potent anti-tumor immunity by inducing extracellular Hsp70-dependent MDSC differentiation. Sci. Rep..

[14] Zhu J., Lou Y., Liu P., Xu L.X. (2020). Tumor-related HSP70 released after cryo-thermal therapy targeted innate immune initiation in the antitumor immune response. Int. J. Hyperthermia Off. J. Eur. Soc. Hyperthermic Oncol. N. Am. Hyperth. Group.

[15] He K., Jia S., Lou Y., Liu P., Xu L.X. (2019). Cryo-thermal therapy induces macrophage polarization for durable anti-tumor immunity. Cell Death Dis..

[16] Peng P., Lou Y., Wang J., Wang S., Liu P., Xu L.X. (2022). Th1-Dominant CD4(+) T Cells Orchestrate Endogenous Systematic Antitumor Immune Memory After Cryo-Thermal Therapy. Front. Immunol..

[17] Sade-Feldman M., Yizhak K., Bjorgaard S.L., Ray J.P., de Boer C.G., Jenkins R.W., Lieb D.J., Chen J.H., Frederick D.T., Barzily-Rokni M. (2018). Defining T Cell States Associated with Response to Checkpoint Immunotherapy in Melanoma. Cell.

[18] Ohtsuka S., Ogawa S., Wakamatsu E., Abe R. (2016). Cell cycle arrest caused by MEK/ERK signaling is a mechanism for suppressing growth of antigen-hyperstimulated effector T cells. Int. Immunol..

[19] Zumwalde N.A., Domae E., Mesher M.F., Shimizu Y. (2013). ICAM-1-dependent homotypic aggregates regulate CD8 T cell effector function and differentiation during T cell activation. J. Immunol..

[20] Zenke S., Palm M.M., Braun J., Gavrilov A., Meiser P., Böttcher J.P., Beyersdorf N., Ehl S., Gerard A., Lämmermann T. (2020). Quorum Regulation via Nested Antagonistic Feedback Circuits Mediated by the Receptors CD28 and CTLA-4 Confers Robustness to T Cell Population Dynamics. Immunity..

[21] Shan J., Jing W., Ping Y., Shen C., Han D., Liu F., Liu Y., Li C., Zhang Y. (2024). LFA-1 regulated by IL-2/STAT5 pathway boosts antitumor function of intratumoral CD8(+) T cells for improving anti-PD-1 antibody therapy. Oncoimmunology.

[22] Gazdik T.R., Crow J.J., Lawton T., Munroe C.J., Theriault H., Wood T.M., Albig A.R. (2024). Notch intracellular domains form transcriptionally active heterodimeric complexes on sequence-paired sites. Sci. Rep..

[23] Minter L.M., Turley D.M., Das P., Shin H.M., Joshi I., Lawlor R.G., Cho O.H., Palaga T., Gottipati S., Telfer J.C. (2005). Inhibitors of γ-secretase block in vivo and in vitro T helper type 1 polarization by preventing Notch upregulation of Tbx21. Nat. Immunol..

[24] Ross S.H., Cantrell D.A. (2018). Signaling and Function of Interleukin-2 in T Lymphocytes. Annu. Rev. Immunol..

[25] Ligocki A.J., Brown J.R., Niederkorn J.Y. (2016). Role of interferon-γ and cytotoxic T lymphocytes in intraocular tumor rejection. J. Leukoc. Biol..

[26] Zimmerman M., Yang D., Hu X., Liu F., Singh N., Browning D., Ganapathy V., Chandler P., Choubey D., Abrams S.I. (2010). IFN-γ upregulates survivin and Ifi202 expression to induce survival and proliferation of tumor-specific T cells. PLoS ONE.

[27] Pipkin M.E., Sacks J.A., Cruz-Guilloty F., Lichtenheld M.G., Bevan M.J., Rao A. (2010). Interleukin-2 and inflammation induce distinct transcriptional programs that promote the differentiation of effector cytolytic T cells. Immunity.

[28] Shouse A.N., LaPorte K.M., Malek T.R. (2024). Interleukin-2 signaling in the regulation of T cell biology in autoimmunity and cancer. Immunity.

[29] Li H., Liu H., Liu Y., Wang X., Yu S., Huang H., Shen X., Zhang Q., Hong N., Jin W. (2023). Exploring the dynamics and influencing factors of CD4 T cell activation using single-cell RNA-seq. Science.

[30] Mutascio S., Mota T., Franchitti L., Sharma A.A., Willemse A., Bergstresser S.N., Wang H., Statzu M., Tharp G.K., Weiler J. (2023). CD8(+) T cells promote HIV latency by remodeling CD4(+) T cell metabolism to enhance their survival, quiescence, and stemness. Immunity.

[31] Verma N.K., Fazil M.H., Ong S.T., Chalasani M.L., Low J.H., Kottaiswamy A., Kizhakeyil A., Kumar S., Panda A.K., Freeley M. (2016). LFA-1/ICAM-1 Ligation in Human T Cells Promotes Th1 Polarization through a GSK3β Signaling-Dependent Notch Pathway. J. Immunol..

[32] Harjunpää H., Llort Asens M., Guenther C., Fagerholm S.C. (2019). Cell Adhesion Molecules and Their Roles and Regulation in the Immune and Tumor Microenvironment. Front. Immunol..

[33] Shi H., Shao B. (2023). LFA-1 Activation in T-Cell Migration and Immunological Synapse Formation. Cells.

[34] Gérard A., Cope A.P., Kemper C., Alon R., Köchl R. (2021). LFA-1 in T cell priming, differentiation, and effector functions. Trends Immunol..

[35] Sabatos C.A., Doh J., Chakravarti S., Friedman R.S., Pandurangi P.G., Tooley A.J., Krummel M.F. (2008). A synaptic basis for paracrine interleukin-2 signaling during homotypic T cell interaction. Immunity.

[36] Gérard A., Khan O., Beemiller P., Oswald E., Hu J., Matloubian M., Krummel M.F. (2013). Secondary T cell-T cell synaptic interactions drive the differentiation of protective CD8+ T cells. Nat. Immunol..

[37] Chirathaworn C., Kohlmeier J.E., Tibbetts S.A., Rumsey L.M., Chan M.A., Benedict S.H. (2002). Stimulation through intercellular adhesion molecule-1 provides a second signal for T cell activation. J. Immunol..

[38] Edlund K., Madjar K., Mattsson J.S.M., Djureinovic D., Lindskog C., Brunnström H., Koyi H., Brandén E., Jirström K., Pontén F. (2019). Prognostic Impact of Tumor Cell Programmed Death Ligand 1 Expression and Immune Cell Infiltration in NSCLC. J. Thorac. Oncol. Off. Publ. Int. Assoc. Study Lung Cancer.

[39] Chen C., Zou P., Wu X. (2024). Development and Validation of an Immune Prognostic Index Related to Infiltration of CD4+ and CD8+ T Cells in Colorectal Cancer. Mol. Biotechnol..

[40] Palomero J., Panisello C., Lozano-Rabella M., Tirtakasuma R., Díaz-Gómez J., Grases D., Pasamar H., Arregui L., Duch E.D., Fernández E.G. (2022). Biomarkers of tumor-reactive CD4(+) and CD8(+) TILs associate with improved prognosis in endometrial cancer. J. Immunother. Cancer.

[41] Peng P., Lou Y., Wang S., Wang J., Zhang Z., Du P., Zheng J., Liu P., Xu L.X. (2022). Activated NK cells reprogram MDSCs via NKG2D-NKG2DL and IFN-γ to modulate antitumor T-cell response after cryo-thermal therapy. J. Immunother. Cancer.

[42] Hickman A., Koetsier J., Kurtanich T., Nielsen M.C., Winn G., Wang Y., Bentebibel S.-E., Shi L., Punt S., Williams L. (2022). LFA-1 activation enriches tumor-specific T cells in a cold tumor model and synergizes with CTLA-4 blockade. J. Clin. Investig..

