# Supplementary material for: LFA-1/ICAM-1 Interactions Between CD8+ and CD4+ T Cells Promote CD4+ Th1-Dominant Differentiation and CD8+ T Cell Cytotoxicity for Strong Antitumor Immunity After Cryo-Thermal Therapy
Source: Cells. 2025 Apr 21;14(8):620. doi: 10.3390/cells14080620 (PMC12025417; doi:10.3390/cells14080620)
Supplement: Supplementary file 1 [file cells-14-00620-s001.zip › Supplementary Materials.pdf]

Supplementary Materials

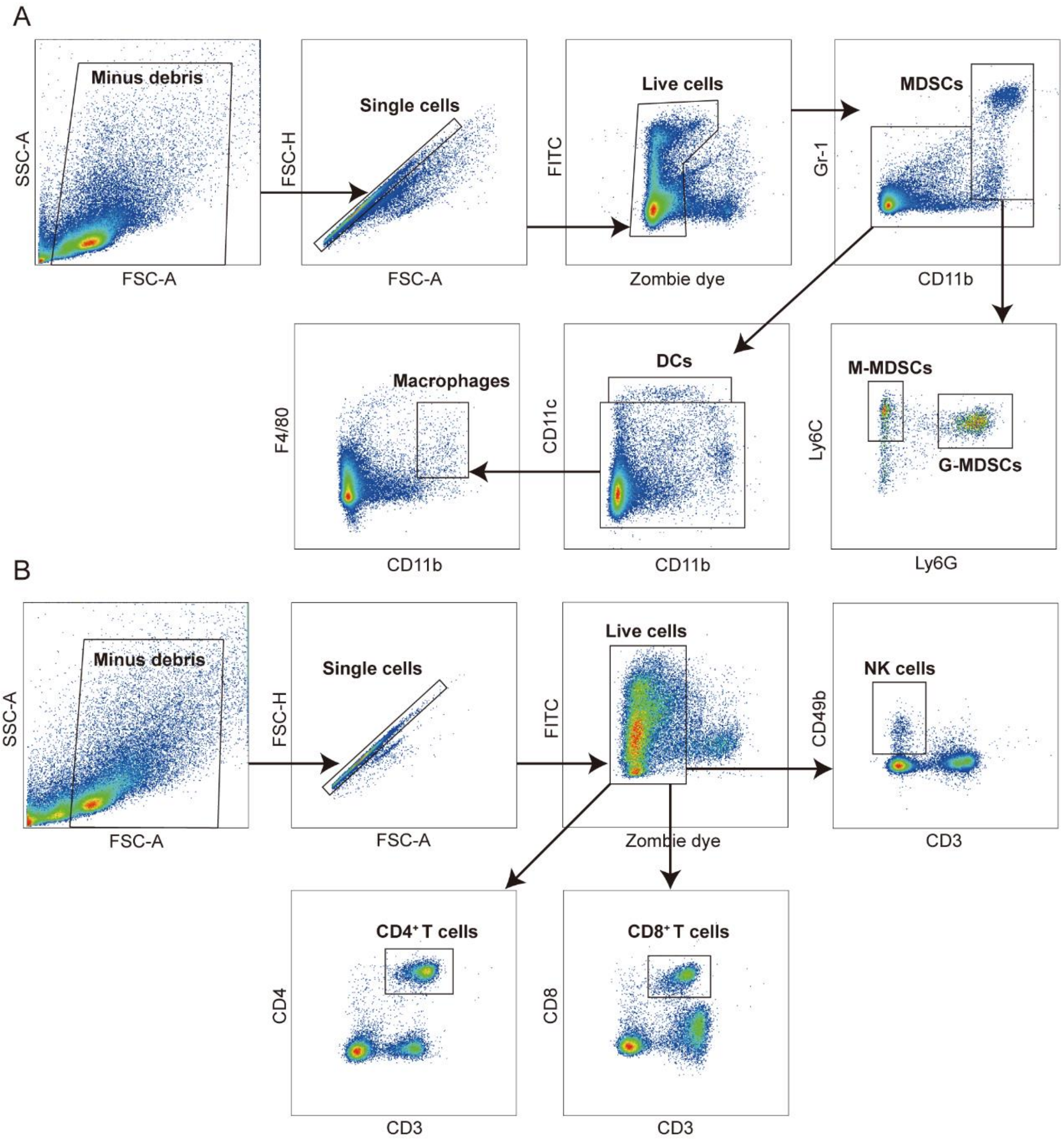

Figure S1 Gating strategy of flow cytometry. (A) The gating strategy of myeloid cells and (B) T cells, NK cells.

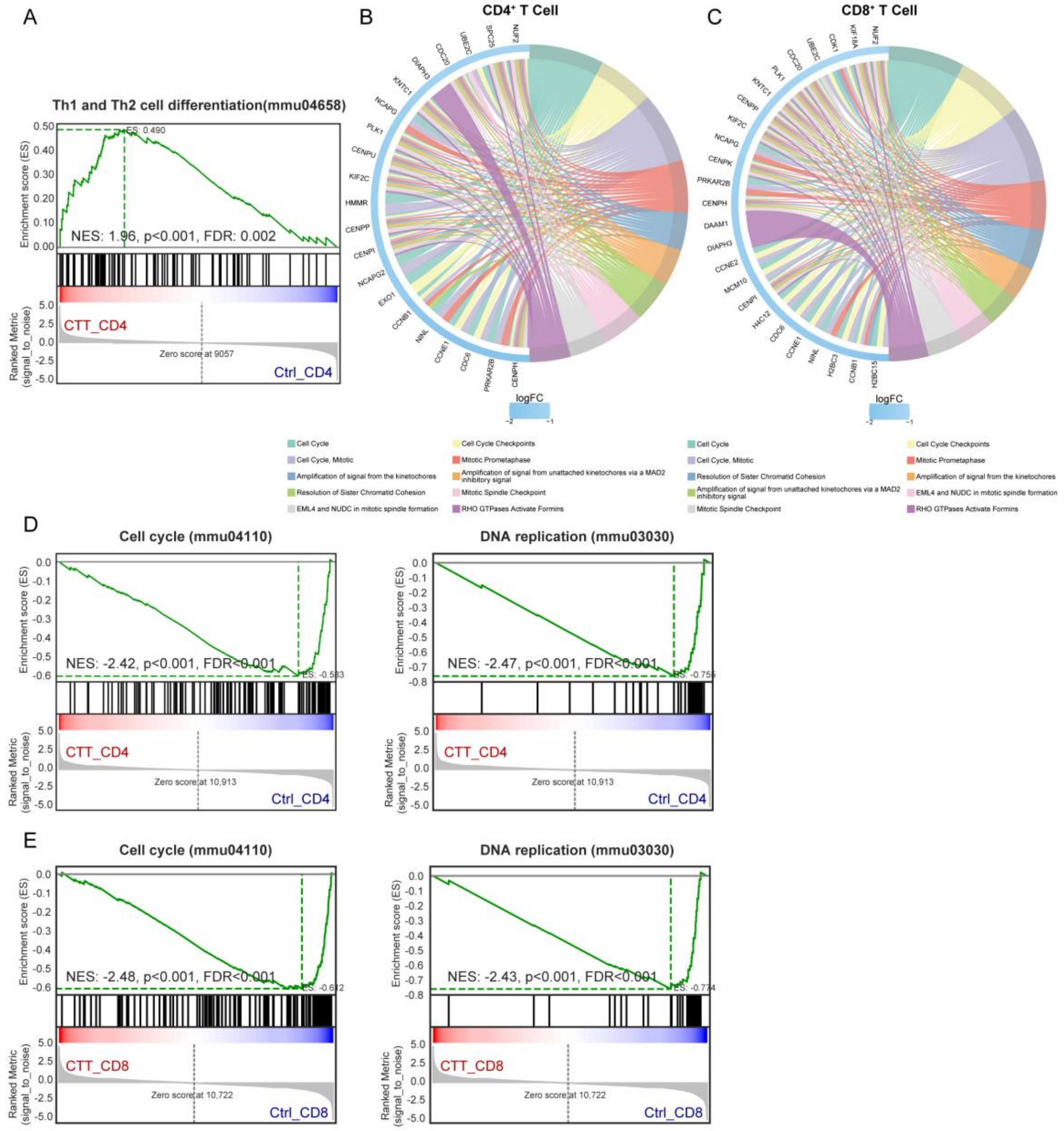

Figure S2 GSEA of CD4^{+} and CD8^{+} T cells in the tumor-bearing control and CTT groups. (A) GSEA of Th1 and Th2 cell differentiation in CD4^{+} T cells based on KEGG. (B-C) The core network with the top 10 downregulated KEGG pathways in enrichment analysis in (B) CD4^{+} and (C) CD8^{+} T cells. (D-E) GSEA of cell cycle pathway and DNA replication pathway based on KEGG terms in (D) CD4^{+} and (E) CD8^{+} T cells. n=3 for RNA-seq.

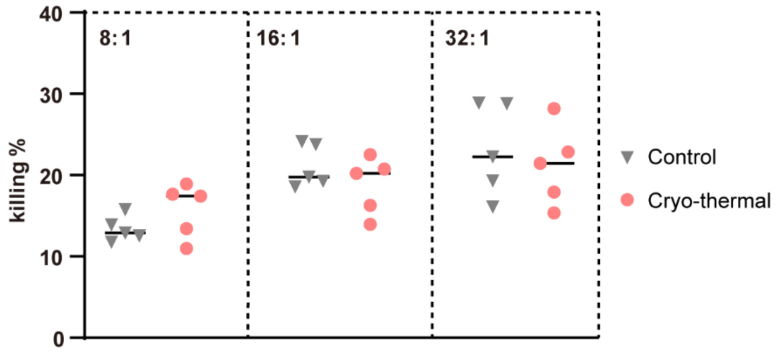

Figure S3 The tumor killing capacity of CD8$^+$ T cells from the tumor-bearing control and CTT-treated mice. n=5 for each group. Data for graphs were calculated by Student's *t*-test.

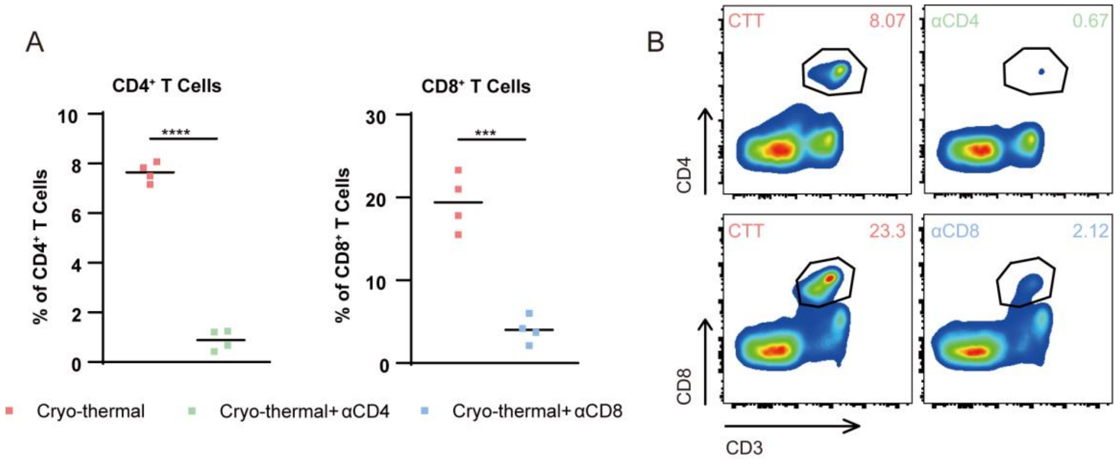

Figure S4 The percentage of $CD4^+$ and $CD8^+$ T cells after T-cell depletion. (A) The violin plots and (B) FACS representative plots of the proportion of $CD4^+$ and $CD8^+$ T cells in the CTT and T-cell depletion groups on day 14 after CTT. All of the data are presented as the means ± SD. n=4 for each group. *** $p < 0.001$, **** $p < 0.0001$. Data for graphs were calculated by Student's *t*-test.

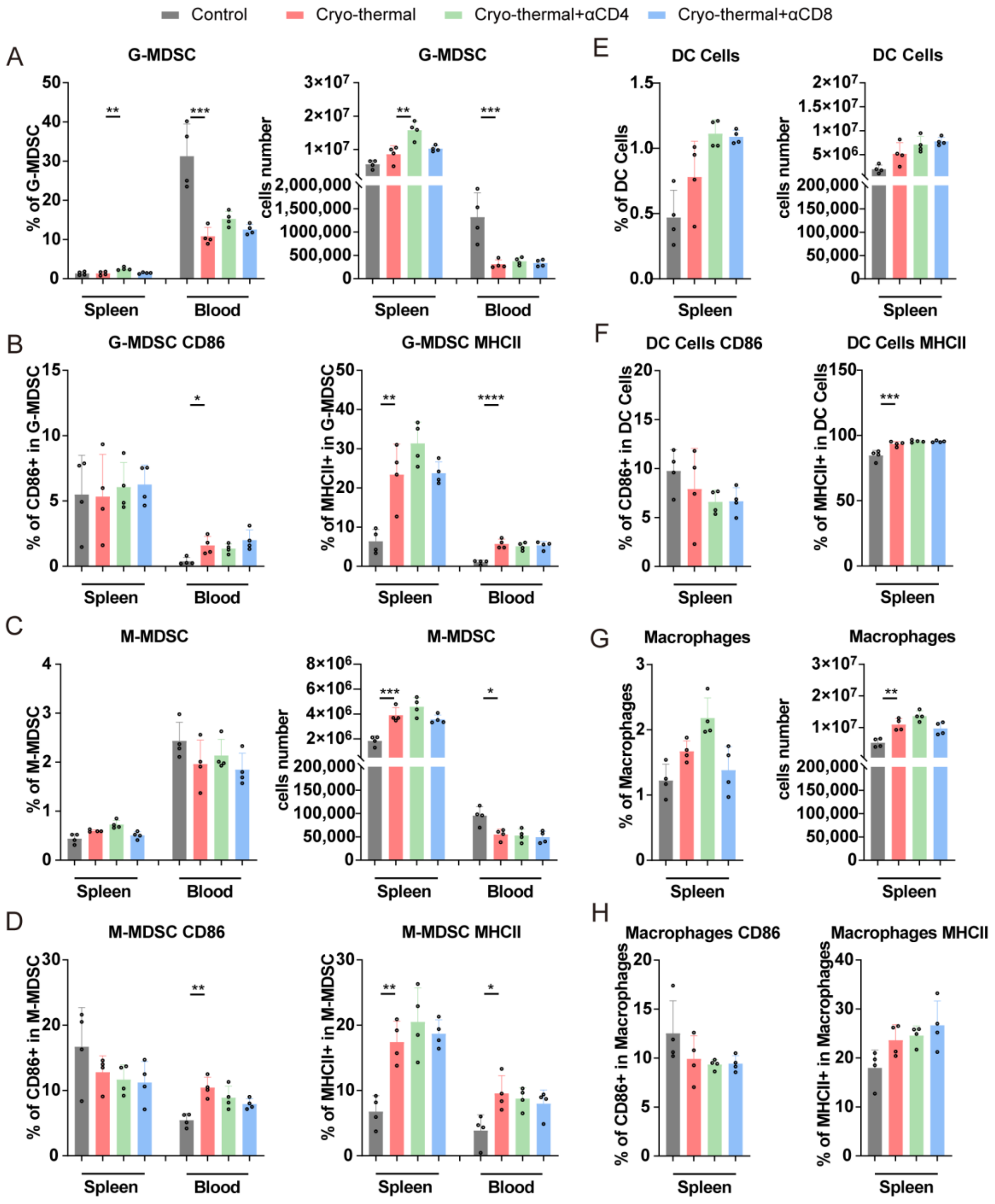

Figure S5 The amount and phenotype of myeloid cells under depletion of T cells after CTT in vivo. (A) The percentage and absolute number of G-MDSC. (B) The expression of CD86 and MHC II on G-MDSC. (C) The percentage and absolute number of M-MDSC. (D) The expression of CD86 and MHC II on M-MDSC. (E) The percentage and absolute number of DCs. (F) The expression of CD86 and MHC II on DCs. (G) The percentage and absolute number of macrophages. (H) The expression of CD86 and MHC II on macrophages. All of the data are presented as the means ± SD. n=4 for each group. * $p < 0.05$, ** $p < 0.01$, *** $p < 0.001$, **** $p < 0.0001$. Data for graphs were calculated by one-way ANOVA.

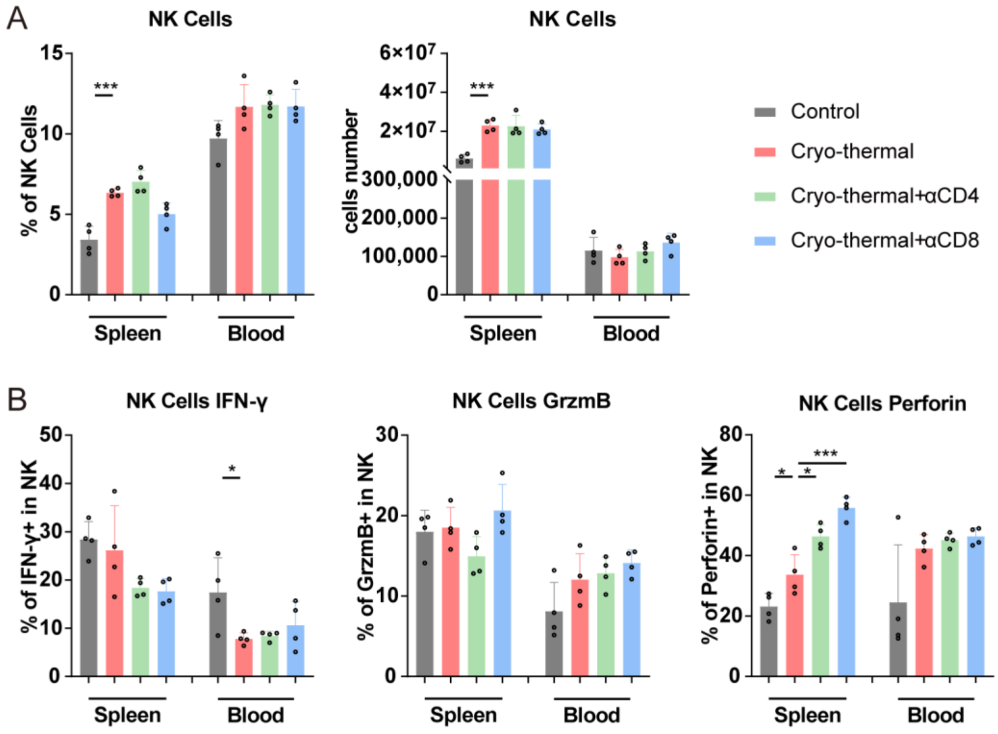

Figure S6 The amount and phenotype of NK cells under depletion of T cells after CTT in vivo. (A) The percentage and absolute number of NK cells. (B) IFN-γ, granzyme B and perforin expression in NK cells. All of the data are presented as the means ± SD. n=4 for each group. * $p < 0.05$, *** $p < 0.001$. Data for graphs were calculated by one-way ANOVA.

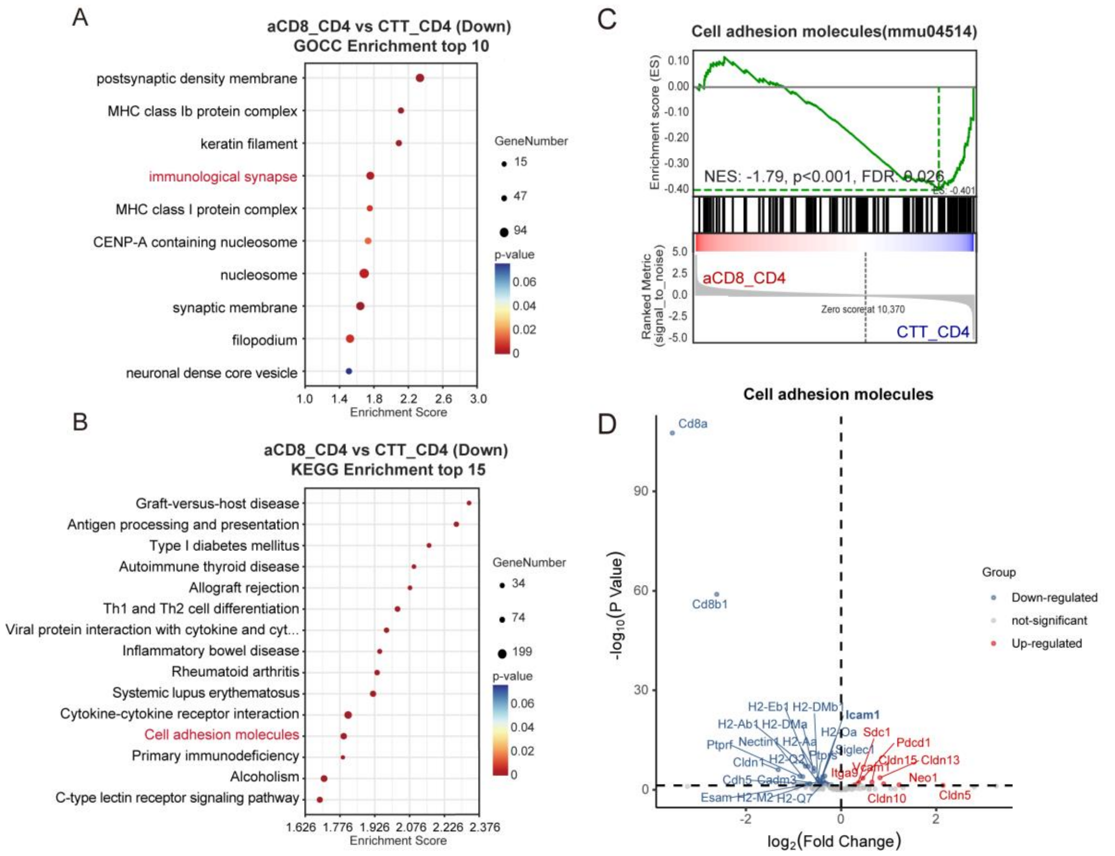

Figure S7 GSEA of $CD4^+$ T cells in the CTT and CTT with $CD8^+$ T-cell depletion groups. (A) Scatter plot of the top 10 downregulated GO enriched gene sets according to GSEA in $CD4^+$ T cells. (B) Scatter plot of the top 15 downregulated KEGG enriched gene sets according to GSEA in $CD4^+$ T cells. (C) Gene enrichment of cell adhesion molecules pathway according to GSEA in $CD4^+$ T cells. (D) Volcano plot of cell adhesion molecules pathway in $CD4^+$ T cells. n=3 for RNA-seq.

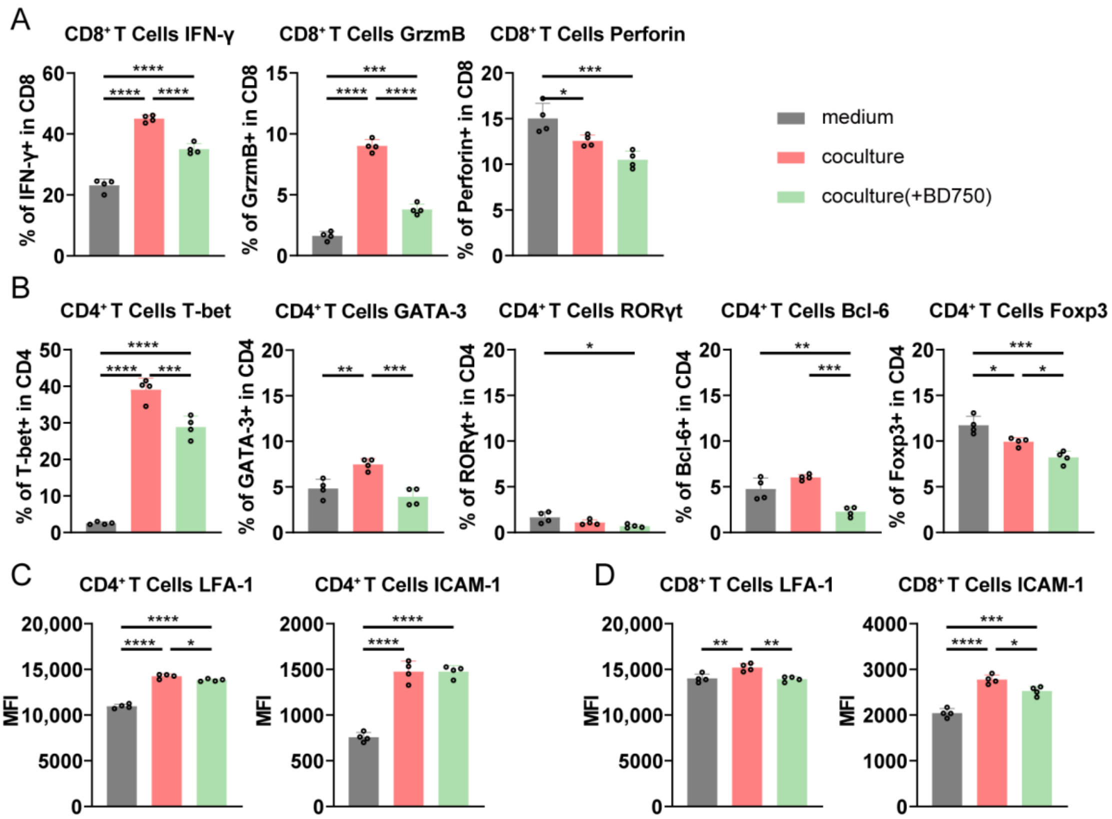

Figure S8 The role of STAT5 in $CD4^+$ and $CD8^+$ T cells interaction. (A) IFN-γ, granzyme B and perforin expression in $CD8^+$ T cells. (B) Subsets of $CD4^+$ T cells. (C-D) The mean fluorescence intensity of LFA-1 and ICAM-1 on (C) $CD4^+$ and (D) $CD8^+$ T cells. All of the data are presented as the means ± SD. n=4 for each group. * $p < 0.05$, ** $p < 0.01$, *** $p < 0.001$, **** $p < 0.0001$. Data for graphs were calculated by one-way ANOVA.

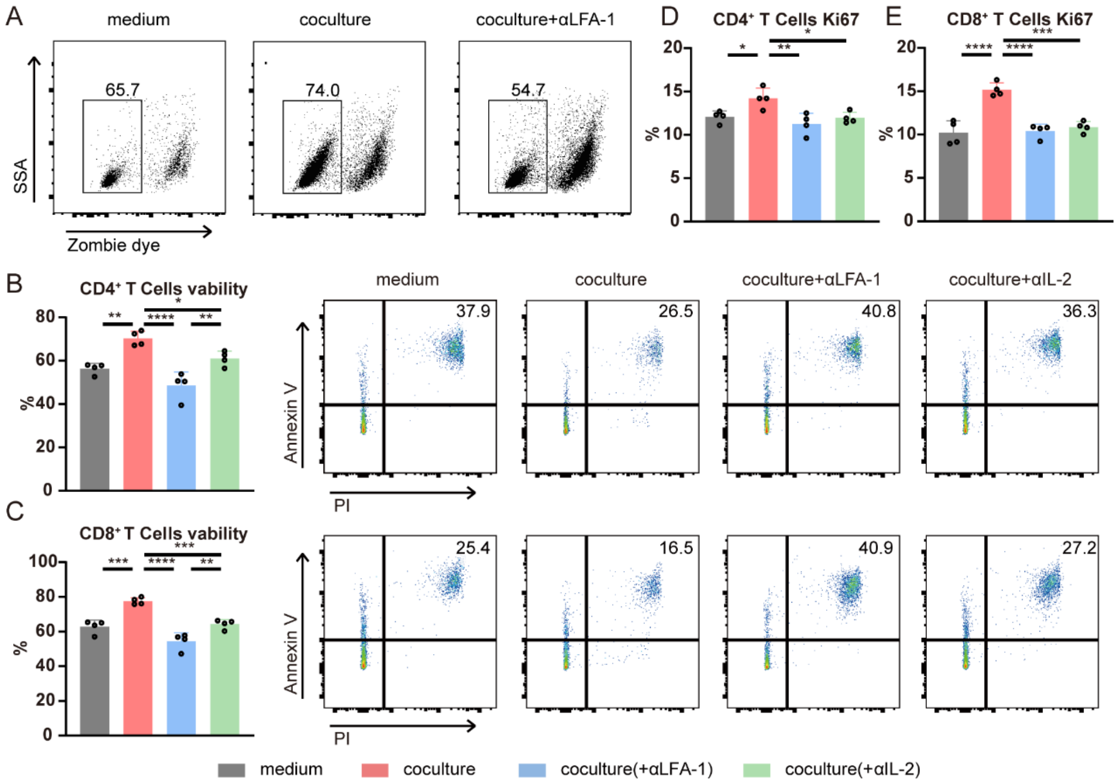

Figure S9 The LFA-1/ICAM-1-IL-2 axis between CD4$^+$ and CD8$^+$ T cells promote the viability and proliferation of T cells. (A) The live cells in different group. (B-C) The viability of (B) CD4$^+$ and (C) CD8$^+$ T cells. (D-E) The expression of Ki67 in (D) CD4$^+$ and (E) CD8$^+$ T cells. All of the data are presented as the means ± SD. n=4 for each group. * $p < 0.05$, ** $p < 0.01$, *** $p < 0.001$, **** $p < 0.0001$. Data for graphs were calculated by one-way ANOVA.

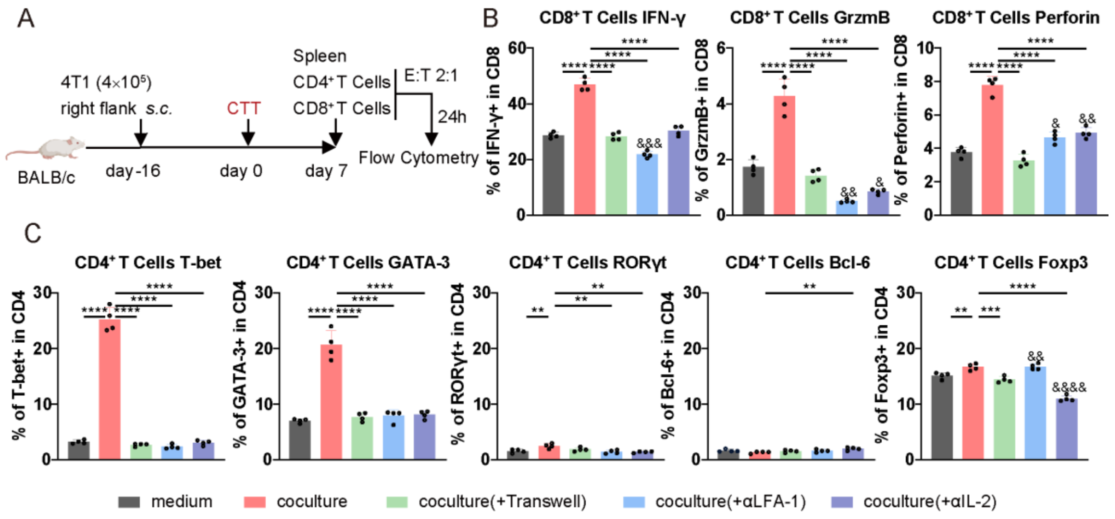

Figure S10 The phenotype of CD4$^+$ and CD8$^+$ T cells in 4T1 tumor model. (A) Scheme of research design. CD4$^+$ and CD8$^+$ T cells were separately isolated by MACS on day 7 after CTT, and then cocultured at an effector-to-target ratio of 2:1 for 24 h. (B) IFN-γ, granzyme B and perforin expression in CD8$^+$ T cells. (C) Subsets of CD4$^+$ T cells. All of the data are presented as the means ± SD. n=4 for each group. * $p < 0.05$, ** $p < 0.01$, *** $p < 0.001$, **** $p < 0.0001$. Compared with the medium group, $^{\&}$ $p < 0.05$, $^{\&\&}$ $p < 0.01$, $^{\&\&\&}$ $p < 0.001$, $^{\&\&\&\&}$ $p < 0.0001$. Data for graphs were calculated by one-way ANOVA.

Table S1 Antibodies used for flow cytometry

Antibodies	Fluorescence Labeling	Clone	Company
CD11b	Pacific Blue	M1/70	Biolegend
CD11c	PE	N418	Biolegend
Gr1	APC	RB6-8C5	Biolegend
Ly6C	FITC	HK1.4	Biolegend
Ly6G	PE/Cy7	1A8	Biolegend
F4/80	BV711	BM8	Biolegend
CD86	APC/Cy7	GL-1	Biolegend
I-A/I-E	PerCP/Cy5.5	M5/114.15.2	Biolegend
CD3	APC/Cy7	145-2C11	Biolegend
CD4	APC or PE/Cy7	RM4-5	Biolegend
CD8	AF700	53-6.7	Biolegend
NK1.1	PerCP/Cy5.5	PK136	Biolegend
T-bet	BV605	4B10	Biolegend
GATA3	AF488	16E10A23	Biolegend
RORγt	BV786	Q31-378	BD
Bcl-6	BV421	K112-91	BD
Foxp3	PE	MF-14	Biolegend
IFN-γ	BV605	XMG1.2	Biolegend
granzyme B	FITC	GB11	Biolegend
perforin	PE	S16009A	Biolegend
IL-4	BV421	11B11	Biolegend
IL-17A	BV711	TC11-18H10.1	Biolegend
IL-21	APC	FFA21	eBioscience
Ki67	BUV395	B56	Biolegend
CD11a/CD18	PE	H155-78	Biolegend
CD54	FITC	YN1/1.7.4	Biolegend
IL-2	APC	JES6-5H4	BD
